# Enhancing the Stability of Pt/Carbon with Ti_3_C_2_ MXene-Derived TiO_2_ for the Oxygen Reduction Reaction in Acidic Media

**DOI:** 10.1021/jacs.6c09621

**Published:** 2026-09-28

**Authors:** Mehmet Turan Görüryilmaz, Bastian Schmiedecke, Aline Alencar Emerenciano, Iris Dorbandt, Karuppasamy Dharmaraj, Thorsten Schultz, Xuyun Guo, Bing Wu, Jiri Sturala, Namrata Sharma, Michael Haumann, Christian Göllner, Karl J. J. Mayrhofer, Zdenek Sofer, Norbert Koch, Valeria Nicolosi, Tristan Petit, Michelle P. Browne

**Affiliations:** † Department of Catalytic Materials: Design and Integration, 28340 Helmholtz-Zentrum Berlin für Materialien und Energie GmbH, Hahn-Meitner-Platz 1, 14109 Berlin, Germany; ‡ Helmholtz-Zentrum Berlin für Materialien und Energie GmbH, 14109 Berlin, Germany; § Institut für Physik & CSMB, Humboldt-Universität Zu Berlin, 12489 Berlin, Germany; ∥ School of Chemistry, CRANN and AMBER Research Centers, 8809 Trinity College Dublin, Dublin 2 D02W9K7, Ireland; ⊥ Department of Inorganic Chemistry, University of Chemistry and Technology Prague, 16628 Prague, Czech Republic; # Helmholtz Young Investigator Group Nanoscale Solid−Liquid Interfaces, Helmholtz-Zentrum Berlin für Materialien und Energie GmbH, Hahn-Meitner-Platz 1, 14109 Berlin, Germany; ∇ Department Atomic-Scale Dynamics in Light-Energy Conversion (PS-ADLU), Helmholtz-Zentrum Berlin für Materialien und Energie GmbH, Hahn-Meitner-Platz 1, 14109 Berlin, Germany; ○ Forschungszentrum Jülich GmbH Helmholtz Institute Erlangen-Nürnberg for Renewable Energy (IET-2), Cauerstr. 1, 91058 Erlangen, Germany

## Abstract

Platinum on carbon remains the proton-exchange membrane fuel cell cathode state-of-the-art catalyst; however, durability losses tied to support corrosion and Pt coarsening hinder this catalyst. This study investigates utilizing different variations of Vulcan XC-72 and Ti_3_C_2_ MXene to improve the oxygen-reduction ORR activity and stability. Material characterization results provide in-depth knowledge of the Pt, carbon, and MXene composites. X-ray photoelectron spectroscopy of the synthesized samples shows that the Ti_3_C_2_ has been oxidized, and high-resolution transmission electron microscopy further reveals that the Pt(111) fringes are present with local anatase TiO_2_ derived from Ti_3_C_2_ MXene, which is also confirmed by X-ray microscopy, indicating that the active support is a Ti_3_C_2_-derived TiO_2_/carbon support. After a U.S. Department of Energy-derived accelerated stress test (AST), the Pt/75%C+25%Ti_3_C_2_ catalyst outperforms an in-house-made Pt/C prepared by the same method. This improvement is accompanied by reduced Pt particle growth after the AST for the Pt/75%C+25%Ti_3_C_2_ when compared to the Pt/C, which is attributed to stabilization of Pt at the MXene-derived anatase surface through Pt–O–Ti bonds, consistent with interfacial anchoring, together with the greater corrosion resistance of the oxide, in a composition that retains sufficient carbon for electronic percolation and gas transport. This study is the first coupled cathode half-cell performance and potential-cycling durability testing for MXene-derived composites benchmarked against Pt/C and provides vital information on material discovery utilizing MXenes for the ORR.

## Graphical abstract

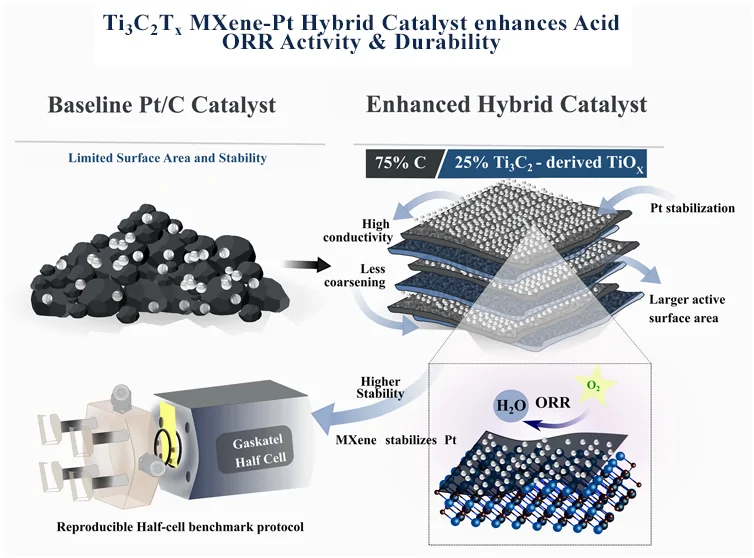

## Introduction

Proton-exchange membrane fuel cells (PEMFCs) rely on platinum (Pt) as an electrocatalyst at the cathode due to the high activity toward the slow oxygen reduction reaction (ORR) kinetics, which can operate with stability under harsh acidic conditions.^1,2^ ORR overpotential on Pt comes from the binding energy compromise across intermediates, which means that tuning the electronic structure of the Pt is more important than changing the mechanism.^3^

Commercially, Pt/C is the industry standard as it is the best-performing ORR material and has many advantages such as carbon’s conductivity and low cost, as well as its ability to disperse Pt well.^2,4^ However, Pt/C loses surface area and activity over time due to carbon corrosion at high cathode potentials or local fuel starvation.^5,6^ Pt also dissolves and ripens under cycling, which further lowers the electrochemically active surface area (ECSA) and shifts size distributions.^7^ These issues make the support the weak link for long-term durability, which means that a better support type is crucial for a longer lifetime. The support type can influence Pt’s specific activity even without changing its particle size or shape by modifying the Pt–support interactions, through the alteration of Pt’s d-band center and oxophilicity.^3^ Because of this, the support chemistry and surface states matter as much as the Pt compositions and morphology.

MXenes are atomically thin layers of transition metal carbides, nitrides, or carbonitrides that are synthesized from their MAX-phase precursor. They are represented by the general formula M_*n*+1_AX*_n_*, where M is an earlier transition metal, A is usually a group 13 or 14 element, and X represents carbon or nitrogen. MXenes combine an attractive surface and compositional area ideal to boost synergetic effects with other elements. In particular, Ti_3_C_2_T_X_ is an attractive support due to its conductivity, hydrophilicity, and well-tunable surface terminations represented by T_X_ in its chemical formula M_*n*+1_X*_n_* T_X_ (−O, −OH, −F, etc.), which can be tailored for optimized metal–support interactions and interfacial wetting.^8,9,10^ Ti_3_C_2_T_X_ can be produced with controlled etching, delamination, and surface chemistry, which allows balancing conductivity, surface oxygen content, the number of layers, and flake size for catalyst inks for energy conversion applications.^10^ However, bare Ti_3_C_2_T_X_ can oxidize under anodic polarization in acid. It has been shown that partial substitution of Ti with tantalum (Ta) in the transition metal (M) layer of the MXene shifts the onset of oxidation to more anodic potentials, extending the potential window over which Ti_3_C_2_T_X_ remains stable above 0 V vs the reversible hydrogen electrode.^11^

Beyond electrochemical conditions, Ti_3_C_2_T_X_ is also chemically and thermally sensitive. In the presence of an oxidant, it converts to TiO_2_ and carbon, and this oxidation is strongly accelerated at elevated temperatures.^12,13^ Hydrothermal treatment of Ti_3_C_2_ in water even at moderate temperatures yields anatase TiO_2_ together with disordered carbon.^14^ The resulting Ti_3_C_2_-derived TiO_X_/carbon material is closely related to the TiO_2_-carbon supports widely studied for the ORR, where oxide domains improve cathode durability.^15,16^ A further oxidation route arises from the reducing character of Ti_3_C_2_ itself, which can donate electrons to reducible metal ion species and become oxidized in the process.^17^

Another important consideration when evaluating any new ORR catalyst is benchmarking the catalyst under protocols comparable to those of the industry. The rotating disk electrode (RDE) method is sensitive to ink formulation, film thickness, Pt loading on the glassy carbon (GC) disk, potential scales, and mass transport corrections.^2,18^ Inconsistent protocols can give multifold differences for the same Pt/C-coated electrode, so reporting and benchmarking must follow strict procedures,^19,20^ However, even good and consistent RDE metrics do not translate into membrane-electrode assemblies (MEAs). Therefore, half-cell methods that operate at higher current density and realistic gas transport, including gas diffusion electrode (GDE) testing, help bridge the gap to device conditions.^21^

Furthermore, accelerated stress tests (ASTs) are essential because they compress the thousands of operating hours required to evaluate proton-exchange membrane fuel cell (PEMFC) durability into practical laboratory protocols, enabling rapid, mechanism-specific screening of materials and MEAs and standardized benchmarking across programs. Performance decline is quantified by metrics such as mass activity loss and support stability, providing go/no-go criteria and comparability across laboratories.^22^ According to the DoE, it is important to reach the goal of less than 10% performance loss at the end of 5000 h by 2030, ultimately 30,000 h by 2050.

Zhang et al.^23^ dispersed Pt on accordion-like Ti_3_C_2_ (20 wt % Pt) and, in 0.5 M H_2_SO_4_, reported an improved half-wave potential compared to Pt/C in an RDE cell, with an acidic current retention of 89.9–93.6% after 1500 cycles against 70.6–85.6% for Pt/C and notably smaller Pt coarsening (up to 5.49 nm vs 8.86 nm for Pt/C). Xu et al.^4^ self-assembled a CNT-Ti_3_C_2_ hybrid support for Pt and achieved roughly 3.4 times higher mass activity than that of Pt/C with much lower ECSA loss (6%) after 2000 cycles in the RDE compared to Pt/C (27%). Zhang et al.^24^ designed a sandwich-structured Ti_3_C_2_/carbon support for Pt that limited MXene restacking and delivered higher ORR activity, only 1% loss with the composite in half-wave potential compared to 16 mV for Pt/C, and stability tests showed 3.05% loss in mass activity for the composite compared with 36.8% for Pt/C in the RDE. These results suggest that mixing carbon and Ti_3_C_2_ in one catalyst layer can couple the strengths of each material: carbon’s percolation and low density and MXene’s strong metal–support interaction (SMSI) and surface functionality. Notably, all prior Pt/MXene- and Pt/TiO_2_-based durability data were obtained in RDE or MEA configurations; coupled half-cell (GDE) activity and potential-cycling durability for a Ti_3_C_2_-derived composite have not been reported, which allows for more realistic results closer to a fuel cell device.

Parallel improvements have been reported for Pt supported on TiO_2_- and TiO_X_-carbon composites, where the benefit is consistently traced to strong metal–support interaction (SMSI). Liu et al.^25^ showed that adding 20 mass % TiO_2_ to Pt/C raised the ORR kinetic current density by 20.5% and halved the degradation rate after SO_2_ poisoning relative to Pt/C, attributing the gain to the Pt–TiO_2_ interaction and the abundant hydroxyl groups on the hydrophilic, electrochemically stable oxide. Chen et al.^26^ engineered a TiO_X_/C support with abundant oxygen vacancies; the vacancies were found to anchor highly dispersed Pt nanoparticles and to strengthen the SMSI through electron transfer between Pt and TiO_X_, yielding electron-rich Pt, enhanced ORR activity, and markedly higher activity retention after accelerated durability testing, whereas Pt/C decayed sharply. Pham et al.^27^ conducted operando near-ambient pressure X-ray photoelectron spectroscopy (XPS) on Pt/TiO_X_/C and provided direct evidence of this effect, showing that the partially reduced TiO_X_ layer in the SMSI contact with Pt strongly suppresses Pt oxidation at high potentials, with the residual oxidation originating only from Pt deposited on carbon rather than on TiO_X_. At the support level, Ayyubov et al.^28^ demonstrated that mixed Ti_(1–X)_Mo_X_O_2_–carbon composites deliver higher Pt stability than that of plain carbon, since the electrocorrosion-resistant TiO_2_ skeleton suppresses support and dopant dissolution. Across these studies, the recurring theme is that a TiO_2_/TiO_X_ phase in intimate contact with Pt anchors the particles, modulates their electronic structure through SMSI, and resists carbon-type corrosion, thereby improving durability.

Beyond charge transfer, TiO_2_ can stabilize supported Pt by binding it more strongly at under-coordinated surface sites: density functional calculations give per-atom Pt binding energies on anatase that rise from 2.87 eV on the stoichiometric (101) surface to 5.05 eV at reduced, under-coordinated surface sites, so that Pt nucleates preferentially at these sites and resists migration and coalescence.^29^ Scanning-tunneling and first-principles studies of anatase (101) confirm that Pt monomers bind at terrace, step, and defect sites strongly enough to suppress sintering.^30^ The oxide additionally protects the carbon support: at the 1.0–1.5 V transient potentials generated by fuel starvation and start–stop events, carbon corrodes rapidly,^31^ whereas a TiO_2_ interlayer roughly halves the electrochemical surface area loss of Pt/C under high-potential cycling by covering reactive carbon sites and limiting their contact with water.^32^ This protection is possible because TiO_2_ is thermodynamically passive across a wide potential window in acid and far more corrosion-resistant than carbon at these potentials.^33^ Finally, the polymorph that forms matters: anatase has a markedly lower surface energy than that of rutile (0.4 versus 2.2 J m^–2^) and is the thermodynamically stable phase for TiO_2_ crystallites below ∼14 nm, which is why moderate-temperature oxidation of Ti_3_C_2_ yields predominantly nanoscale anatase.^34^ It should be noted, however, that such geometric and electronic contributions are not cleanly separable. They are generally understood to act together, as different manifestations of the same underlying metal–support interaction, and a recent critical review cautions that the various MSI subclassifications are difficult to assign and can be misleading in practice.^35^

Despite these promising results, three gaps remain in MXene-based composites for fuel cells. First, the carbon-to-Ti_3_C_2_ ratio has not been varied systematically to map the trade-off between carbon’s conductivity, percolation, and oxidation-prone but Pt-anchoring chemistry of Ti_3_C_2_ in realistic conditions. Second, the consequences of Ti_3_C_2_ oxidation for the ORR activity and durability are rarely quantified. Third, durability for these supports has been assessed almost exclusively by the RDE rather than under the higher current, realistic mass transport conditions of a half-cell. In this study, Pt/C, Pt/Ti_3_C_2_, and three Pt/C+Ti_3_C_2_ compositions are synthesized and benchmarked under a DoE AST-based protocol in GDL flow setups to address both support effects relevant for the industry and allow for protocol-robust benchmarking of the MXene-based composites compared to the in-house-made Pt/C.

## Results and Discussion

PEMFCs suffer from slow ORR kinetics and the instability of the Pt/C electrocatalyst. Improving performance and, more importantly, the long-term stability of the catalysts can facilitate the progress toward widespread industrial applications. We present our material characterization and electrochemical evaluation results on the synthesized Pt electrocatalyst with carbon, Ti_3_C_2_, or C/Ti_3_C_2_ at different ratios as support structures.

The synthesis of Pt on different support types was achieved via facile solvothermal synthesis, as given in Figure 1a. Before the Pt electrocatalyst synthesis, Ti_3_C_2_ MXene was prepared by etching of the MAX phase (Ti_3_AlC_2_) to remove the Al layer and obtain multilayered Ti_3_C_2_. After that, multilayered Ti_3_C_2_ was sonicated in a bath, leading to delaminated Ti_3_C_2_ flakes. Pt/C+Ti_3_C_2_ composites were synthesized using different ratios of delaminated Ti_3_C_2_ and carbon at 180 °C for 8 h. Pt/C and Pt/Ti_3_C_2_ were also synthesized for comparison. The details of the synthesis route are given in the experimental section.

**Figure 1:**
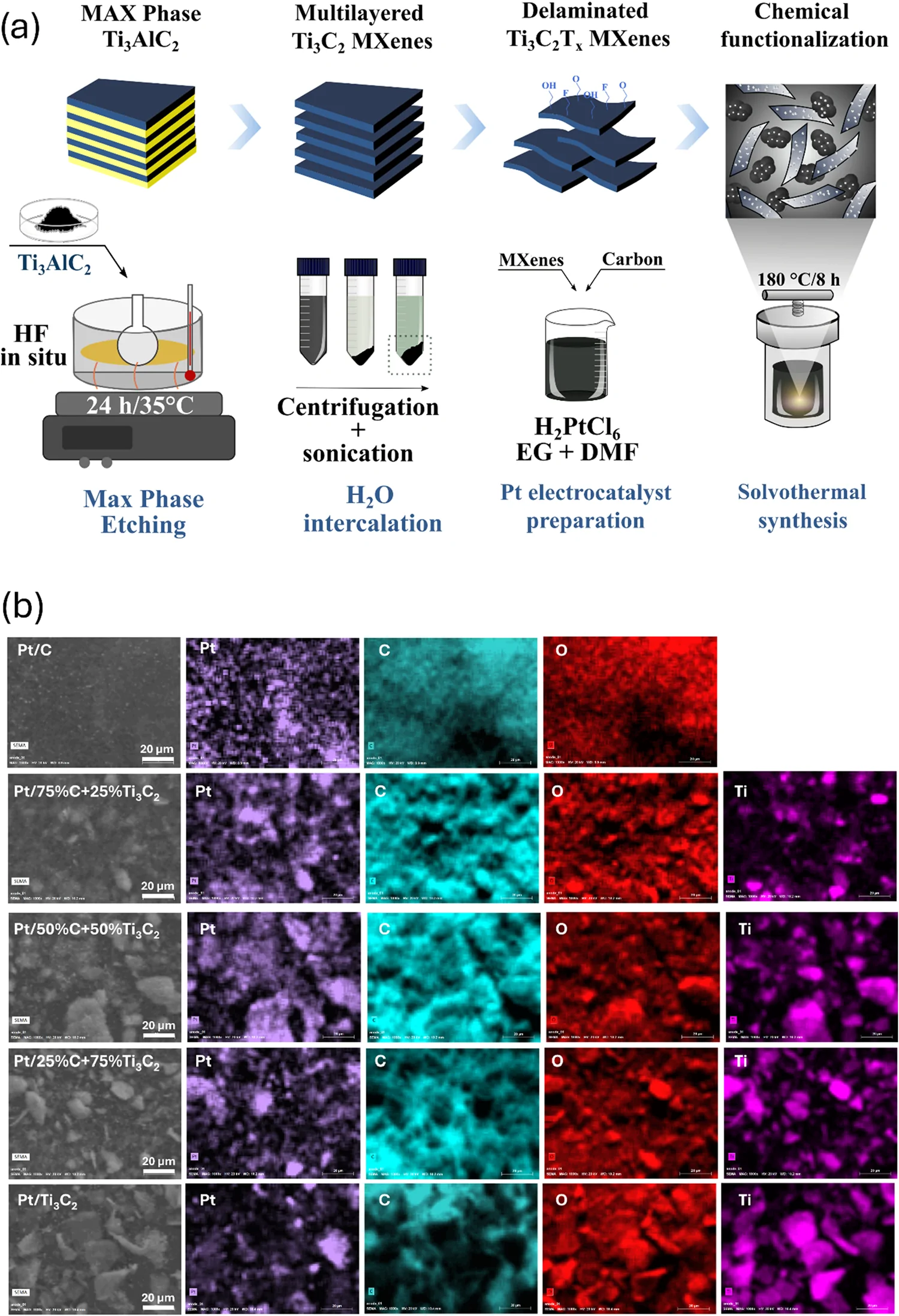
(a) Schematic representation of the Pt/C+Ti_3_C_2_ synthesis process. (b) SEM-EDX maps (Pt, Ti, C, O): Pt mostly colocalized with the support in composites; weaker Pt–Ti overlap for Pt/Ti_3_C_2_; hotspots match Pt particles.

The morphological structures of the catalysts were examined through scanning electron microscopy (SEM), as given in Figure SI2. SEM revealed large (up to 20 μm) and thin Ti_3_C_2_ flakes. For all samples, Pt is distributed on the support uniformly, except for the Pt/Ti_3_C_2_ as Ti and Pt maps do not align uniformly, which can be seen in Figure 1b. Pt clusters up to 100 nm were observed on all samples; however, the amount of these clusters increases with the increasing amount of Ti_3_C_2_ in the sample. The smaller, individual Pt nanoparticles can be seen as blurry white dots in the images due to their small size, which makes it difficult to observe in SEM.

The crystalline structures of the synthesized catalysts were examined through X-ray diffraction (XRD). Figure 2a,b shows the XRD spectra for the thin-film Ti_3_C_2_ MXene (1 μm-thick), as well as the synthesized electrocatalysts in the powder form. The absence of the MAX-phase peaks at 9.48°, 19°, 33.9°, 36.8°, 38.7°, 41.6°, 48.3°, and 56.3° indicates the successful removal of Al and the formation of Ti_3_C_2_. The presence of the characteristic (002) peak of the MXene can be seen clearly at 7.14° in the Ti_3_C_2_ thin film. For the powder composites, the (002) peak of the MXene cannot be observed. Vulcan XC-72 shows two broad reflections, a strong (002) band centered near ∼25°, and a weaker (100)/(101) band near 43°, characteristic of carbon with disordered, small stacking domains (Figure SI3). The anatase TiO_2_ (101) reflection can be seen at 25° for the MXene-derived TiO_2_-containing samples. A minor reflection at 27° is also observed; although its position is close to rutile TiO_2_ (110), no rutile is detected by SAED and XAS, so it is assigned to a minor MXene-derived Ti suboxide. Another anatase reflection peak (105) at 53° can be observed for the MXene-derived TiO_2_-containing samples. The anatase detected by SAED and HRTEM is therefore present as a thin, nanocrystalline/flake-bound phase, consistent with its weak XRD signature. Crystalline Pt peaks at (111), (200), (220), (311), and (222) confirm the successful Pt synthesis for all the samples. The sample holder peaks are given in Figure SI3; the broad band between 25° and 35° is the result of the holder.

**Figure 2:**
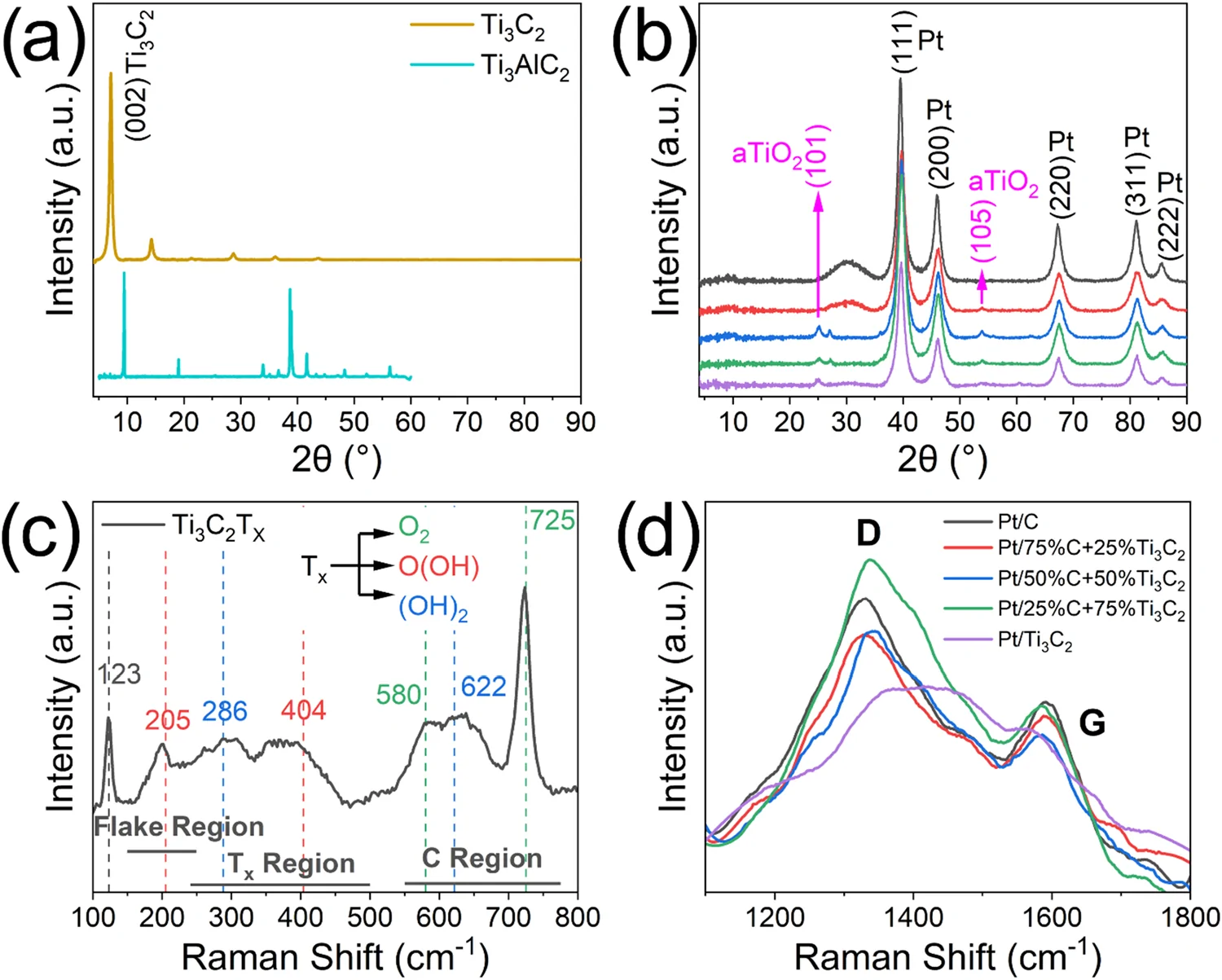
XRD spectra of (a) MAX-phase Ti_3_AlC_2_ and Ti_3_C_2_ MXene and (b) synthesized electrocatalysts. Raman spectra of (c) Ti_3_C_2_ flakes and (d) synthesized electrocatalysts.

Figure 2c presents the Raman shifts of Ti_3_C_2_ and its terminal groups. Both Ti and C atoms yield E_g_ (in plane) and A_1g_ (out of plane) peaks. Because of this, the spectra can be examined in three regions. The first one is the flake region, made of two titanium layers and functional groups, as well as a group vibration of carbon. The T_*x*_ region corresponds to the vibrations of surface groups; in-plane and out-of-plane vibrations are found in the carbon region (C region). A sharp Ti peak was observed at 123 cm^–1^. The surface termination on the flakes mostly consists of O_2_ (580 cm^–1^ E_g_ peak and 725 cm^–1^ A_1g_ peak, both for C), O­(OH) (205 cm^–1^ A_1g_ peak for Ti and 404 cm^–1^ E_G_ peak for Ti), and (OH)_2_ (286 cm^–1^ E_g_ peak for Ti and 622 cm^–1^ A_1g_ peak for C) species.^36^

Figure 2d compares the carbon D and G bands across composites. At 785 nm wavelength excitation, the D band (∼1325–1347 cm^–1^) and G band (∼1582–1590 cm^–1^) with no resolvable D’ (∼1620 cm^–1^) can be observed. The crystallite sizes are ordered as Pt/75%C+25%Ti_3_C_2_ > Pt/C > Pt/50%C+50%Ti_3_C_2_ > Pt/25%C+75%Ti_3_C_2_ > Pt/Ti_3_C_2_ according to the *L*_a_ ≈ 91.1 (*I*_G_/*I*_D_) nm formula. The MXene-only spectrum is not interpretable by this metric since it does not contain graphitic carbon. The G-band full width at half maximum (fwhm) is largest for the Pt/50%C+50%Ti_3_C_2_ sample, while Pt/25%C+75%Ti_3_C_2_, Pt/75%C+25%Ti_3_C_2_, and Pt/C show comparably narrower and similar G band widths. The D band fwhm ranks Pt/25%C+75%Ti_3_C_2_, followed by Pt/C, with the narrowest D bands for Pt/75%C+25%Ti_3_C_2_ and Pt/50%C+50%Ti_3_C_2_, which are similar to each other. These trends indicate increasing edge/defect density with MXene addition but highlight that excessive broadening (especially in the D band) accompanies highly disordered/amorphous contributions.

Bright-field transmission electron microscopy (TEM) shows Pt nanoparticles uniformly distributed on the support, with no large agglomerates (Figure 3). High-resolution TEM (HRTEM) images resolve lattice fringes that were indexed to fcc Pt (111); this assignment to fcc Pt (111) matches the Pt (111) XRD reflection at ∼39.8°. At selected areas of the support, localized fringes consistent with anatase TiO_2_ (101) appear at the surface; Ti 2p XPS also indicates oxidation to anatase TiO_2_.

**Figure 3:**
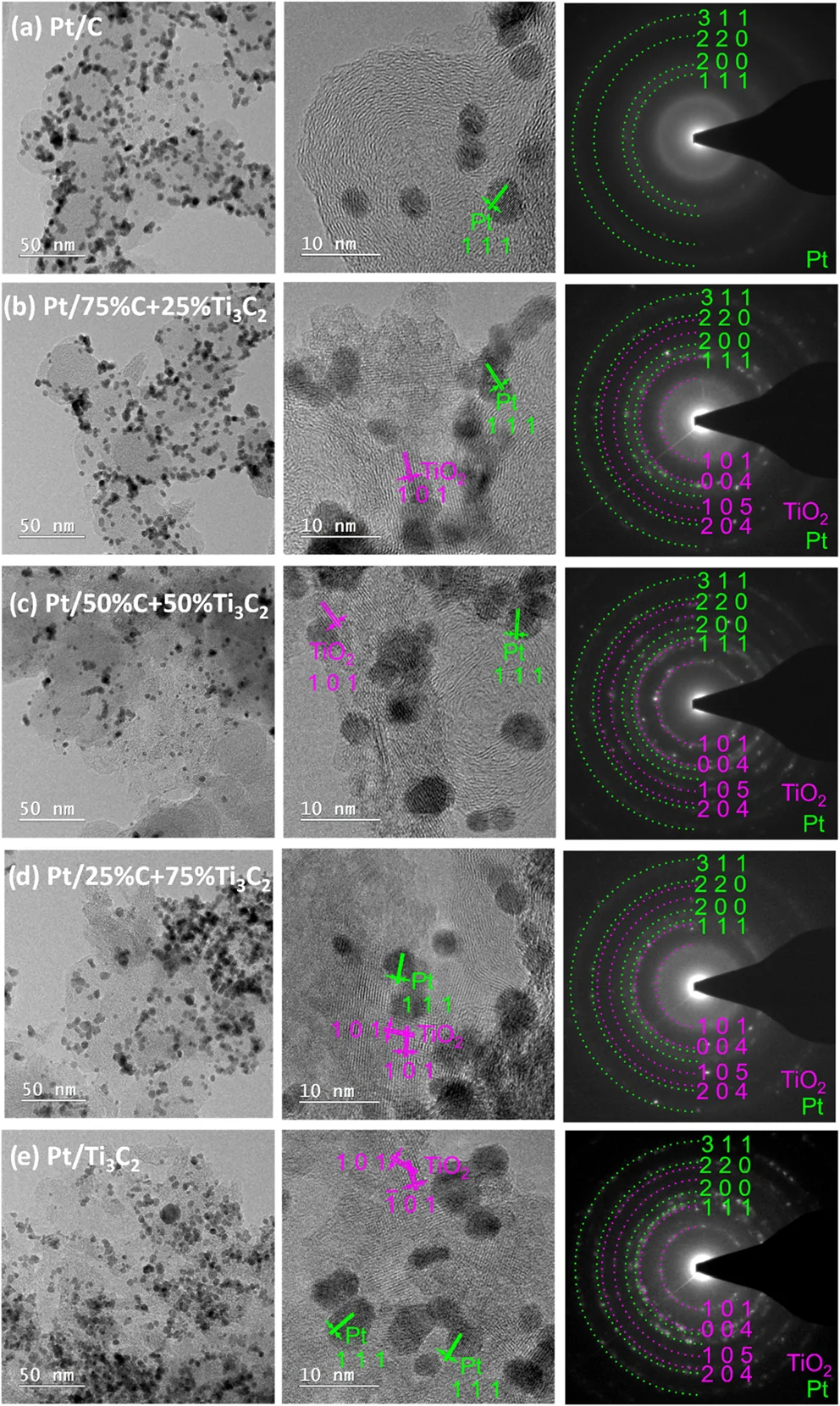
TEM and HRTEM images of a representative particle with Pt (111) fringes and the support with fringes indexed as anatase TiO_2_ (101) and SAED patterns indicating the Pt and TiO_2_ *rings*.

Bright-field TEM confirms that Pt nanoparticles are deposited on the supports in all compositions and are predominantly present as discrete particles across the imaged regions. In Ti_3_C_2_-containing catalysts, the support retains a flake-like, sheet morphology characteristic of MXene, and STEM elemental maps show Pt colocated with the support framework: Pt overlaps with carbon-rich domains in Pt/C and with Ti-containing regions in the composites, supporting successful Pt deposition throughout the mixed C-Ti_3_C_2_ architecture. Pt nanoparticles are uniformly distributed on the Pt/C sample, with a size distribution centered in the few-nanometer range (mean: 5.3 nm), with most particles lying between 2 and 6 nm (∼68%). SAED patterns exhibit ring reflections that can be indexed to fcc Pt (111), (200), (220), and (311), confirming crystalline metallic Pt.

Pt remains finely dispersed on the mixed support for Pt/75%C+25%Ti_3_C_2_ (mean: 5.2 nm), with the majority of particles in the 2–6 nm window (∼75%). SAED shows fcc Pt rings at (111), (200), (220), and (311). In addition, SAED reveals rings attributable to anatase TiO_2_ at (101), (004), (105), and (204), indicating that Ti_3_C_2_ layers turned into an anatase-like oxide component. Pt particle size distribution remains largely within the few-nanometer regime for Pt/50%C+50%Ti_3_C_2_ (mean: 5.2 nm), with ∼72% of particles between 2 and 6 nm. SAED again confirms fcc Pt via (111), (200), (220), and (311) rings and shows the anatase TiO_2_ ring set ((101), (004), (105), (204)) in the Ti_3_C_2_-containing support, consistent with oxidation of the MXene.

Increasing the Ti_3_C_2_ fraction broadens the Pt size distribution and shifts the mean to a slightly larger value for Pt/25%C+75%Ti_3_C_2_ (mean: 5.7 nm), with ∼66% of particles between 2 and 6 nm and a more visible large-size tail (∼6% above 10 nm). SAED patterns display the same fcc Pt ring sequence ((111), (200), (220), (311)) and, for the Ti_3_C_2_-containing support, anatase TiO_2_ rings ((101), (004), (105), (204)), indicating that Pt retains the same crystalline phase, while the support includes an anatase-like oxide signature.

Pt/Ti_3_C_2_ shows the smallest mean size among the series (mean: 4.8 nm), with most particles between 2 and 6 nm (∼83%) and only a small fraction above 10 nm (∼3%). SAED confirms fcc Pt via (111), (200), (220), and (311) rings, while anatase TiO_2_ rings at (101), (004), (105), and (204) are also observed, demonstrating that Ti_3_C_2_-containing samples consistently exhibit an anatase-like oxide contribution. Together with HRTEM observations of Pt (111) lattice fringes and localized anatase TiO_2_ (101) fringes at the support, these diffraction results indicate that Pt crystallizes as fcc nanoparticles in all samples, whereas Ti_3_C_2_-based supports exhibit oxidation to anatase TiO_2_ without evidence for a new Pt phase introduced by MXene addition.

Overall, the microscopy and diffraction data show that changing the carbon-to-Ti_3_C_2_ ratio primarily influences the Pt particle size distribution and dispersion uniformity, while the Pt crystalline structure remains fcc across all catalysts. The consistent appearance of anatase TiO_2_ rings in all Ti_3_C_2_-containing samples supports a common structural feature of these composites: Ti_3_C_2_-based flakes persist in the catalyst layer but with an anatase-like oxide component detectable by electron diffraction and local HRTEM indexing.

ADF-STEM and the corresponding elemental maps (Figure 4) were used to evaluate nanoscale element distribution in the catalyst particles and to verify how the support composition evolves across the C+Ti_3_C_2_ series. In contrast to SEM-EDX, ADF-STEM EDX and EELS maps indicate a homogeneous Pt nanoparticle distribution in all imaged regions for every sample, including Pt/Ti_3_C_2_, with no evidence of Pt-rich “islands” at the mapping length scale. Across the composite series, the relative carbon signal decreases as Ti_3_C_2_ content increases, while the Ti signal increases accordingly. Oxygen is consistently colocated with Ti and strengthens with increasing Ti_3_C_2_ fraction, consistent with progressive oxidation associated with the Ti-containing support domains.

**Figure 4:**
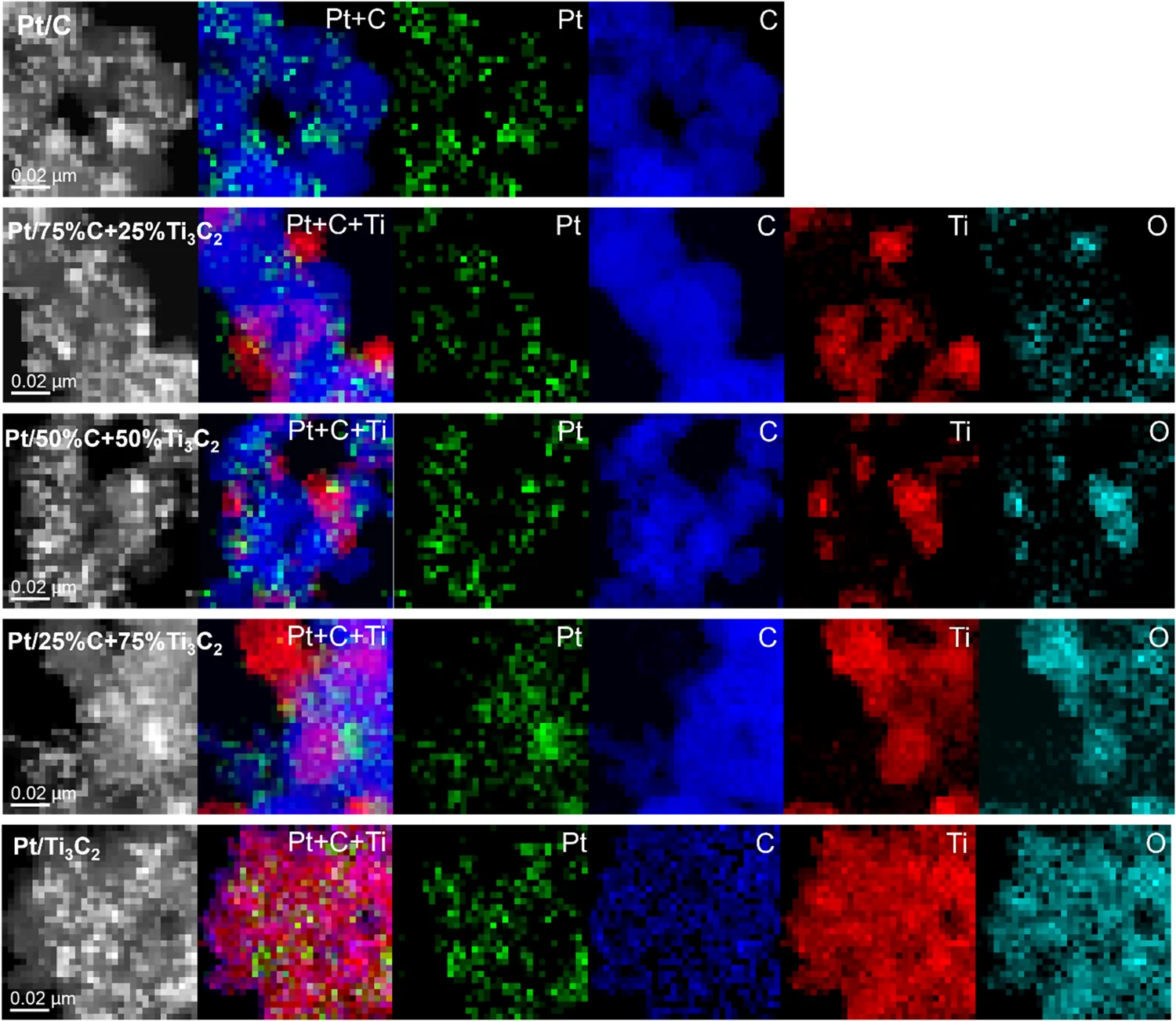
ADF-STEM image and corresponding elemental maps (EDS signal: Pt, EELS signal: Ti, C, O) for powders, matching the TEM fields.

Pt and C maps overlap broadly across the carbon support for Pt/C, indicating uniform Pt dispersion on carbon within the analyzed areas; no Ti or O signal is expected or observed. Pt is uniformly distributed across the mixed support for Pt/75% C+25% Ti_3_C_2_. The Ti map confirms the presence of Ti_3_C_2_ domains, while the oxygen signal remains comparatively faint on Ti, relative to other samples. This suppressed O intensity suggests that carbon-rich coverage in this composition partially masks or limits the detectable oxidation of Ti-containing regions, consistent with carbon providing a shielding effect.

Pt remains homogeneously dispersed across the support for Pt/50%C+50%Ti_3_C_2_. Ti and O signals are stronger than those in Pt/75%C+25%Ti_3_C_2_, and oxygen closely follows the Ti distribution, indicating more prevalent oxidized Ti-containing regions as the Ti_3_C_2_ fraction increases. Pt mapping still shows uniform dispersion, while the C signal becomes noticeably weaker compared to the carbon-richer composites for Pt/25%C+75%Ti_3_C_2_. Ti and colocated O signals are prominent, indicating that Ti-containing (and partially oxidized) support regions dominate the mapped areas at this composition. Pt nanoparticles are also uniformly distributed on the Ti-based support at the nanoscale for Pt/Ti_3_C_2_. The carbon signal is minimal as expected. Ti is strongest in this sample, and oxygen matches Ti spatially, indicating an extensive Ti-associated oxygen signal consistent with an oxidized component of the Ti_3_C_2_-derived support.

Overall, ADF-STEM and elemental mapping results support that Pt dispersion is homogeneous at the nanoscale for all catalysts (including Pt/Ti_3_C_2_), the C and Ti contrast tracks the designed support ratios, and oxygen preferentially follows Ti-rich regions, with the comparatively weaker O signal in Pt/75%C+25%Ti_3_C_2_ suggesting partial carbon coverage that mitigates detectable Ti oxidation in that composition.

XPS was used to determine the atomic concentration and the oxidation states of the samples. The Ti 2p, C 1s, and Pt 4f core levels are shown in Figure 5a. Platinum is present purely as metal for all samples, as indicated by two sharp, asymmetric peaks located at 71.2 and 74.5 eV, without any significant changes in the spectra for different amounts of Ti_3_C_2_ added. While the pristine Ti_3_C_2_ is of excellent quality (no signs of oxidation in the Ti 2p spectrum, a strong Ti–C peak at ∼282 eV in the C 1s spectrum), it is completely oxidized for all mixed samples. The oxidation of the MXene to TiO_2_ could result from two scenarios during the synthesis. The first scenario is due to the reaction conditions during the solvothermal process. The second is that the MXene itself acts as a reducing agent for the reduction of the Pt precursor to the Pt metal.^17^ The reduction of Pt^4+^ to metallic Pt is a stepwise redox process (Pt­(IV) → Pt­(II) → Pt(0)) that necessarily oxidizes an electron donor; in conventional colloidal syntheses, this role is filled by added reductants or by electron-rich solids and polymers such as cellulose, which reduce the metal ions while being oxidized themselves.^37,38,39^ In the absence of a dedicated reductant, the electron-rich Ti_3_C_2_ surface can serve this function, donating electrons to the precursor and being oxidized to TiO_X_ in the process, making an oxygen-rich external atmosphere not required. Although the synthesis is carried out under in an N_2_-saturated DMF/ethylene glycol medium, this precursor-driven redox proceeds even in an inert environment (although the vessel was sealed in air), while residual water in the hygroscopic solvents can additionally oxidize the carbide as a reagent.^13^ The 180 °C solvothermal conditions accelerate both pathways.

**Figure 5:**
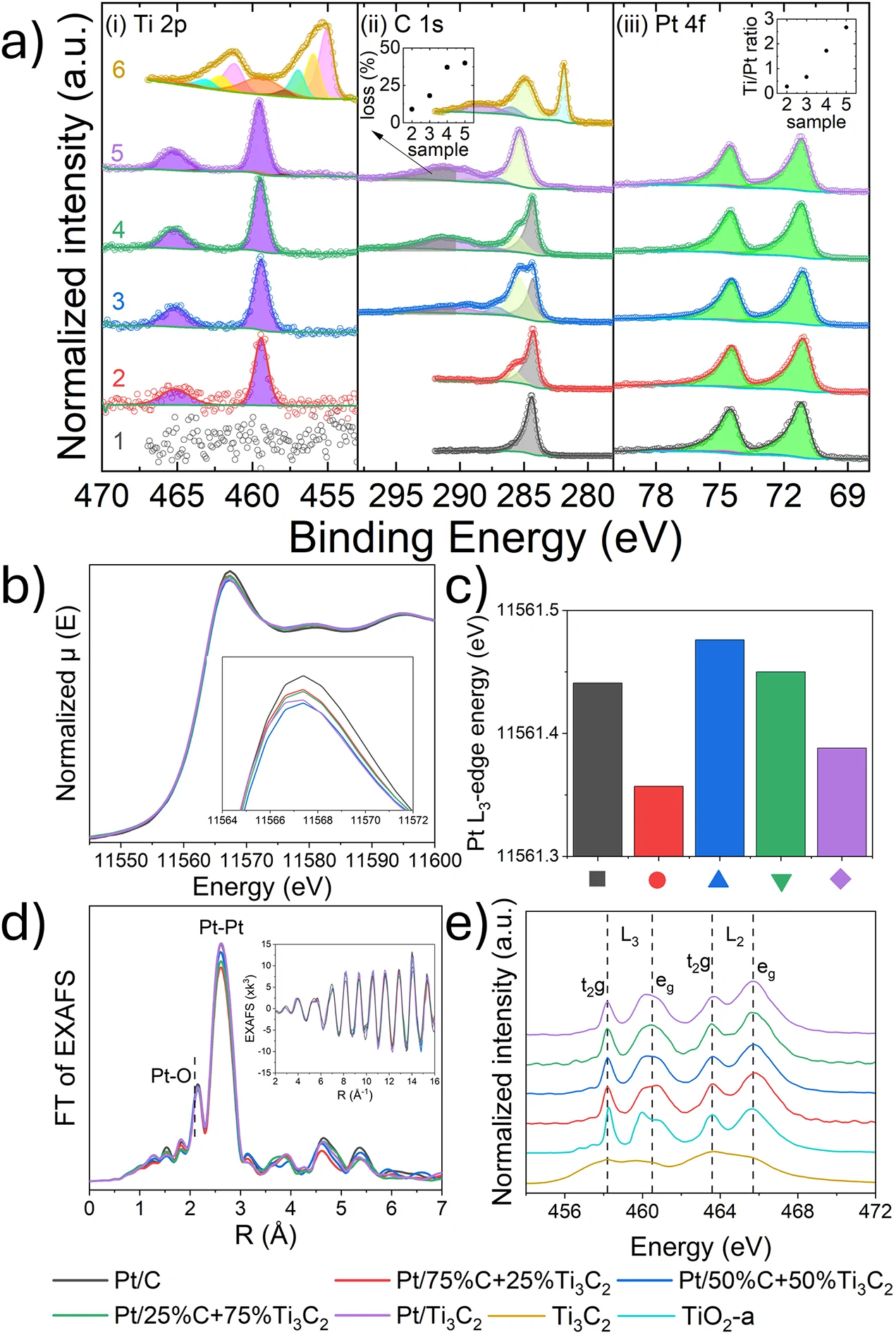
(a) XPS (i) Ti 2p, (ii) C 1s, and (iii) Pt 4f core levels of (1) Pt/C, (2) Pt/75%C+25%Ti_3_C_2_, (3) Pt/50%C+50%Ti_3_C_2_, (4) Pt/25%C+75%Ti_3_C_2_, (5) Pt/Ti_3_C_2_, and (6) bare Ti_3_C_2_. The inset in (ii) shows the ratio of the loss feature, indicated in gray, to the overall C 1s intensity. The inset in (iii) shows the Ti/Pt ratio of samples 2–5. (b) Pt L_3_-edge XANES of the synthesized catalysts (in-house Pt/C and the composite series spanning 0–100% Ti_3_C_2_ support) and magnified white line intensities in the inset. (c) Comparison of Pt L_3_-edge energies (determined by the integral over edge amplitudes in the range of 0.15–0.9). (d) Fourier-transforms of Pt L_3_-edge EXAFS, FTs were calculated for a k-range of 1.6–16.4 Å-1 using cos-windows over 10% at both range ends; the EXAFS spectra are shown in the inset. (e) Ti L_2,3_-edge XAS in total electron yield mode.

The Ti/Pt ratio, shown as the inset in Figure 5a (iii), increases linearly with the added amount of Ti_3_C_2_. We further noticed an increase in intensity in the C 1s spectrum around 292 eV with increasing Ti_3_C_2_ content, which cannot be attributed to oxidation alone (typically in the range of 286–290 eV). C–F_X_ bonds, typically observed at this energy, can be ruled out too, as no fluorine was observed for these samples. Peaks at this energy are often assigned to surface plasmon losses.^40,41^ The interaction of platinum with the Ti_3_C_2_ seems to introduce some sort of energy loss that increases linearly with the Ti/Pt ratio until it saturates at large Ti_3_C_2_ content, as shown in the inset in Figure 5a (ii). The origin of this loss is not clear at the moment, but it correlates with the decrease in performance for larger amounts of Ti_3_C_2_. Inset plots are also given in Figure SI4 on a larger scale.

Across the composition series, the Pt L_3_-edge XANES (Figure 5b,c) shows only small variations in the edge position and white line intensity that do not trend with Ti_3_C_2_-derived TiO_2_ content, and Pt supported on pure Ti_3_C_2_-derived TiO_2_ is spectroscopically indistinguishable from Pt on carbon; the EXAFS (Figure 5d) is dominated by metallic Pt–Pt scattering, within which any minor Pt–Ti contribution is not separately resolvable, and the Pt 4f binding energy is almost invariant in XPS (−0.08 eV shift for only C+MXene mixed samples). These small spectral differences reflect Pt nanoparticle size and a slight degree of surface oxidation common to all supports, rather than metal-to-support charge transfer, so an electronic metal–support interaction between the Pt and Ti can be excluded. This does not exclude an interfacial Pt–support bond. Metal–support interactions encompass structural and enthalpic effects as well as electronic ones,^35^ and the data are consistent with an interfacial anchoring interaction in which Pt is bound at the anatase surface through Pt–O–Ti bonds without charge transfer to or from the metal. Consistent with this, high-resolution MAADF-STEM shows Pt NPs anchored on TiO_2_ nanoflakes (Figure SI5). From the literature, this has been shown by that the DFT-calculated Pt adhesion energy is nearly doubled relative to the ideal surface, pinning the particles against migration and Ostwald ripening without altering their oxidation state.^29,30^ Also, bare TiO_2_ conducts electrons poorly, so too much TiO_2_ (or a thick oxide shell) can limit charge transfer and lower accessible Pt area unless carbon pathways are present.^42^ Previously reported Pt/TiO_2_ cathodes show the advantage of TiO_2_ for the ORR. Liu et al. showed that Pt/TiO_2_/C keeps ∼76% of its initial ECSA after an accelerated aging test, while Pt/C falls to ∼43%, and Pt growth is far less severe on TiO_2_ than on carbon.^15^ Pt/TiO_2_ activity and stability also depend on oxide particle size and Pt loading; for example, 12 wt % Pt on 30 nm TiO_2_ showed the highest ORR activity and only ∼5.9% ECSA loss after accelerated stress test.^43^ Composites that add conductive carbon around TiO_2_ recover percolation and boost performance while keeping the stabilization benefit of the oxide.^16,42^

These trends match our observations. HRTEM shows localized fringes consistent with anatase TiO_2_(101) on the Ti_3_C_2_-derived support, and SAED, Ti L-edge XANES, and Ti 2p XPS together confirm a fully oxidized Ti­(IV) anatase phase.^10^ Because anatase is a corrosion-resistant, nanoscale-stable polymorph, it can survive the potentials that can degrade the support material, and by covering reactive carbon domains, it can slow their oxidation.^32,33^ Overall, a thin, discontinuous anatase layer on Ti_3_C_2_ acts as an anchoring and stabilization motif for Pt, where an overly continuous oxide skin would penalize conductivity; carbon + Ti_3_C_2_ composites appear to offer the best balance for the PEM ORR.

Delaminated Ti_3_C_2_ MXene flakes and Pt/75%C+25%Ti_3_C_2_ hybrid were measured using scanning transmission X-ray microscopy (STXM). Figure SI6a presents the STXM images acquired at the Ti L e_g_ peak energy (463 eV) for both samples. It shows uniform flakes for Ti_3_C_2_ MXene; however, a more distorted flake morphology was observed for Pt/75%C+25%Ti_3_C_2_ hybrid flakes. The electronic structure of both samples at the titanium L-edge and oxygen K-edge is presented in Figure SI6b. The XAS spectra of Ti_3_C_2_ shows characteristic peaks for both Ti L_3_ and L_2_ edges similar to that reported before.^44^ The crystal field splitting parameter, 10Dq (also denoted Δ), was extracted directly from the Ti L-edge XANES spectra by identifying the energy positions of the L_3_ t_2g_ and L_3_ e_g_ absorption peaks. The Ti L_3_ and L_2_ peaks correspond to the transition from Ti 2p_3/2_ and 2p_1/2_ orbitals to the Ti 3d orbital, respectively. In an octahedral ligand field, the Ti 3d orbitals split into a lower energy 3-fold-degenerate t_2g_ set (d_*xy*_, d_*xz*_, d_*yz*_) and a higher energy 2-fold-degenerate e_g_ set (d_*z*_^2^, d_*x*_ *^2^*_– y_^2^), with the energy separation between them defining 10Dq.^44^ Peak positions were determined by locating the local intensity maxima in the first derivative, and 10Dq was calculated as the simple energy difference: 10Dq = E­(L_3_ e_g_) – E­(L_3_ t_2g_). This approach is well-established for Ti L-edge spectroscopy.^45^ The two prominent peaks in the L_3_ manifold correspond directly to transitions from the Ti 2p_3/2_ core level to the unoccupied 3d t_2g_ and e_g_ states. The magnitude of their separation reflects the strength of the local crystal field imposed by the surrounding ligands. The L_3_ t_2g_ (457.50 eV) is shifted to lower energy relative to Pt/75%C+25%Ti_3_C_2_ (457.75 eV), and the L_3_ e_g_ at 459.25 eV shows no splitting, yielding a reduced 10Dq of 1.75 eV consistent with the weaker ligand field of the mixed carbide/surface-termination environment and lower oxidation state of titanium. In the L_2_ region, the t_2g_ at 463.0 eV is well-resolved, but the e_g_ manifests only as a weak shoulder around 464.5 eV, indicating significant broadening and suppression of the octahedral orbital character.

The Pt/75%C+25%Ti_3_C_2_ hybrid exhibits much more prominent splitting for both L_2_ and L_3_ edges with a shoulder to L_3_ e_g_ peak showing spectral features close to the TiO_2_ anatase phase.^46^ The oxidation state of Ti for pristine Ti_3_C_2_ and Pt/75%C+25%Ti_3_C_2_ could also be directly compared by estimating their L_2_e_g_/t_2g_ peak intensity ratio.^47^ Ti has an estimated oxidation of 3.78 for Pt/75%C+25%Ti_3_C_2_ compared to 2.59 for pristine Ti_3_C_2_, corresponding to their L_2_e_g_/t_2g_ peak intensity ratio of 3.78 and 0.79, respectively (Figure SI6c). This suggests that Ti in Pt/75%C+25%Ti_3_C_2_ is significantly more oxidized, approaching +4, than the pristine one.

The O K-edge corroborates this picture: the main absorption peak, reflecting O 2p hybridization with Ti 3d states, shifts systematically from 530.42 eV in Pt/75%C+25%Ti_3_C_2_ to 530.75 eV in Ti_3_C_2_, tracking the change in the Ti–O bonding character from surface termination groups to the rutile phase.^48^ The intermediate position of Pt/75%C+25%Ti_3_C_2_ across all spectral markers (10Dq, e_g_ splitting, L_2_e_g_ resolution, and O K-edge) collectively demonstrates that Pt decoration introduces localized Ti oxidation having an oxidation state close to +4. The intermediate oxidation state (+3.8 rather than +4) and the persistence of flake morphology indicate that the Ti_3_C_2_-derived domains are not fully converted to bulk anatase but remain as an almost completely oxidized flake-bound TiO_2_ phase in electronic contact with the carbon network.

Pt L_3_-edge XANES (Figure 5b and white line comparison in Figure 5c) and EXAFS (Figure 5d) were carried out ex situ for Pt foil, a commercial Pt/C reference, in-house-made Pt/C prepared by the same route as the composites, and the full composite series spanning 0–100% Ti_3_C_2_ in the support. XANES and EXAFS plots for the synthesized materials are shown; the full range of materials including Pt foil and commercial Pt/C are given in Figure SI7. All supported catalysts show edge energies of 0.5–0.9 eV above the foil and modestly enhanced white line intensities, as expected for the nanoparticulate Pt bearing a degree of surface oxidation, with the commercial Pt/C being a clear outlier (+0.9 eV, white line height +0.21 relative to the foil), indicating substantially more oxidized Pt than any of the in-house materials. EXAFS confirms that the composites consist of well-ordered metallic Pt nanoparticles with bulk-like Pt–Pt distances (2.76 Å), Debye–Waller factors indistinguishable from the foil, and first-shell amplitudes of 0.66–0.74 relative to bulk Pt, corresponding to a number-averaged particle size of approximately 2.5 nm; the fitted Pt–O coordination (*N* = 0.6 at 2.09 Å) lies only marginally above the value obtained by fitting the same path to the bulk Pt foil (*N* = 0.3), for which the surface oxide contribution to a bulk-averaged coordination number is negligible (<10^–3^); this therefore represents the effective detection limit for the Pt–O path in this fitting model, far below the genuine oxide contribution of the commercial reference (*N* = 1.9 at 2.00 Å). Critically, none of these parameters varies systematically with the Ti_3_C_2_ content. The edge energy is constant to within 0.1 eV, the white line intensity shows no significant trend (edge energy accuracy: ≤ 0.1 eV), no Pt–Ti scattering contribution can be discriminated from the Pt–Pt distances, and Pt supported on pure Ti_3_C_2_-derived TiO_2_ is spectroscopically indistinguishable from Pt supported on carbon.

The oxidation state and local coordination of the Ti-containing support were probed by Ti L_2,3_-edge XAS in total electron yield (TEY) mode, which samples the outermost ∼5–10 nm and is therefore sensitive to the support surface at which Pt is anchored (Figure 5e). Freeze-dried Ti_3_C_2_ and a crystalline anatase TiO_2_ standard were measured under identical conditions as reduced- and oxidized-Ti references. The Ti_3_C_2_ reference shows the broad, poorly resolved L_3_ envelope characteristic of carbidic Ti, in which the t_2_g and e_g_ features are barely separated. In contrast, all Pt-containing composites display the sharp four-feature multiplet of Ti^4+^ in an oxide environment, with the L_3_ t_2_g at 458.2 eV, the L_3_ e_g_ centroid at 460.5 eV, and the corresponding L_2_ features at 463.7 and 465.8 eV. These energies coincide with those of the anatase reference (t_2_g 458.3 eV, e_g_ 460.5 eV), and no carbidic contribution is detectable in any composite, including the carbon-free Pt/Ti_3_C_2_ sample. Oxidation of the Ti_3_C_2_ precursor to anatase TiO_2_ is therefore complete throughout the probed surface region, independent of support composition, in agreement with the anatase phase identified by SAED, HRTEM, and XPS. The L_3_ t_2_g position and e_g_ centroid are furthermore constant to within 0.08 and 0.05 eV across the full composition series, showing that the Ti oxidation state and local coordination do not change with Ti_3_C_2_ loading. The composite spectra nevertheless differ from crystalline anatase in the degree of multiplet definition. All samples show a higher e_g_/t_2_g intensity ratio (1.02–1.24) than that of the anatase reference (0.89) together with a shallower valley between the t_2_g and e_g_ features, while the peak energies remain unchanged. Because the e_g_/t_2_g ratio is an intensity ratio, it is independent of energy calibration, and the combination of matched peak positions with reduced multiplet resolution is the signature of increased local structural disorder rather than a different Ti phase or oxidation state. Taken with the crystalline anatase observed by SAED, XAS, XPS, and XRD, these data indicate a crystalline anatase core terminated by a structurally disordered surface layer, a morphology that provides both a corrosion-resistant oxide scaffold and the under-coordinated surface sites at which Pt is anchored through Pt–O–Ti bonds. The reason for observing a completely oxidized Ti (+4) in XPS and XAS compared to partially oxidized Ti (+3.8) in STXM is the probing depth difference between the methods. XAS and XPS are surface-sensitive measurements that probe about 10 nm depth, while STXM is a bulk measurement method that can reveal information about the whole flake. STXM averages the fully oxidized surface together with a less-oxidized, carbide-retaining core, yielding a flake-averaged value (+3.8) below the +4 seen by the surface-sensitive probes. The possible presence of carbon-bound Ti in the core of the flake, however, can be neglected in terms of performance as the almost completely oxidized flake (where completely oxidized Ti in the form of TiO_2_ occurs at the surface) will behave like TiO_2_.

Brunauer–Emmett–Teller (BET) analysis to obtain the surface area (Figure SI8) and TGA-DTG analysis to understand the thermal behavior (Figure SI9) of the synthesized materials, as well as their pure counterpats are presented in the SI.

RDE measurements were conducted to observe the intrinsic activity of the catalysts, Figure 6. Figure 6a shows the LSV measurements taken at 1600 rpm with 10 mV/s scan rate in 0.1 M HClO_4_. Both Pt/C and Pt/75%C+25%Ti_3_C_2_ have an onset potential of 1.04 V, while this value is 1.03 V for Pt/50%C+50%Ti_3_C_2_ and 1.02 V for Pt/25%C+75%Ti_3_C_2_. Pt/Ti_3_C_2_ has a lower onset potential of 1.0 V. All the C+Ti_3_C_2_ samples exhibit an elbow at 0.81 V before reaching the diffusion-controlled area at 0.7 V, with values of −5.19 mA/cm^2^, −5.35 mA/cm^2^, −5.6 mA/cm^2^, and −5.75 mA/cm^2^ for Pt/25%C+75%Ti_3_C_2_, Pt/50%C+50%Ti_3_C_2_, Pt/C, and Pt/75%C+25%Ti_3_C_2_, respectively. However, Pt/Ti_3_C_2_ yields a much lower performance compared to the other samples, reaching −3.4 mA/cm^2^ maximum in the diffusion-controlled region. The Tafel slopes of the catalysts are all similar around 60 mV/dec, Figure 6b. Electrochemically active surface area (ECSA) was determined with the area of H_upd_ region from the CV, Figure 6c, curves, scanned with 50 mV/s, Figure 6d. According to the results, the largest ECSA belongs to Pt/75%C+25%Ti_3_C_2_ (38.36 m^2^/g_Pt_), followed by Pt/C (30.53 m^2^/g_Pt_), Pt/50%C+50%Ti_3_C_2_ (17.31 m^2^/g_Pt_), Pt/25%C+75%Ti_3_C_2_ (12.96 m^2^/g_Pt_), and Pt/Ti_3_C_2_ (2.12 m^2^/g_Pt_). This aligns with the smaller surface area seen in the BET surface area results of Ti_3_C_2_ compared to C, which resulted in larger Pt nanoparticle clusters, leading to a smaller active surface. Ti_3_C_2_ alone shows no ORR activity. Mass and specific activities were extracted from *iR*-corrected, mass transport-corrected kinetic currents at 0.9 V vs RHE, normalized to the experimentally determined Pt loading and to the ECSA, respectively. At 0.9 V, Pt/C, Pt/75%C+25%Ti_3_C_2_, Pt/50%C+50%Ti_3_C_2_, Pt/25%C+75%Ti_3_C_2_, and Pt/Ti_3_C_2_ delivered 0.098 ± 0.016 A/mg, 0.143 ± 0.010 A/mg, 0.074 ± 0.012 A/mg, 0.057 ± 0.003 A/mg, and 0.011 ± 0.002 A/mg mass activities, respectively. The specific activities of the samples were calculated as 0.32 ± 0.05 mA/cm^2^, 0.37 ± 0.02 mA/cm^2^, 0.43 ± 0.07 mA/cm^2^, 0.44 ± 0.02 mA/cm^2^, and 0.47 ± 0.08 mA/cm^2^ for Pt/C, Pt/75%C+25%Ti_3_C_2_, Pt/50%C+50%Ti_3_C_2_, Pt/25%C+75%Ti_3_C_2_, and Pt/Ti_3_C_2_, respectively. Half-wave potentials of the samples were also investigated. Pt/C showed 0.913 V, while Pt/75%C+25%Ti_3_C_2_ displayed an improved 0.926 V. Pt/50%C+50%Ti_3_C_2_, Pt/25%C+75%Ti_3_C_2_, and Pt/Ti_3_C_2_ samples have 0.907, 0.898, and 0.834 V, respectively. All comparisons here are made against an in-house-synthesized Pt/C prepared by an identical solvothermal route rather than commercial 40 wt % Pt/C. The in-house Pt/C therefore shows a lower RDE ECSA than typical commercial benchmarks, and absolute activities should be read as internal comparisons under matched synthesis methods and testing.

**Figure 6:**
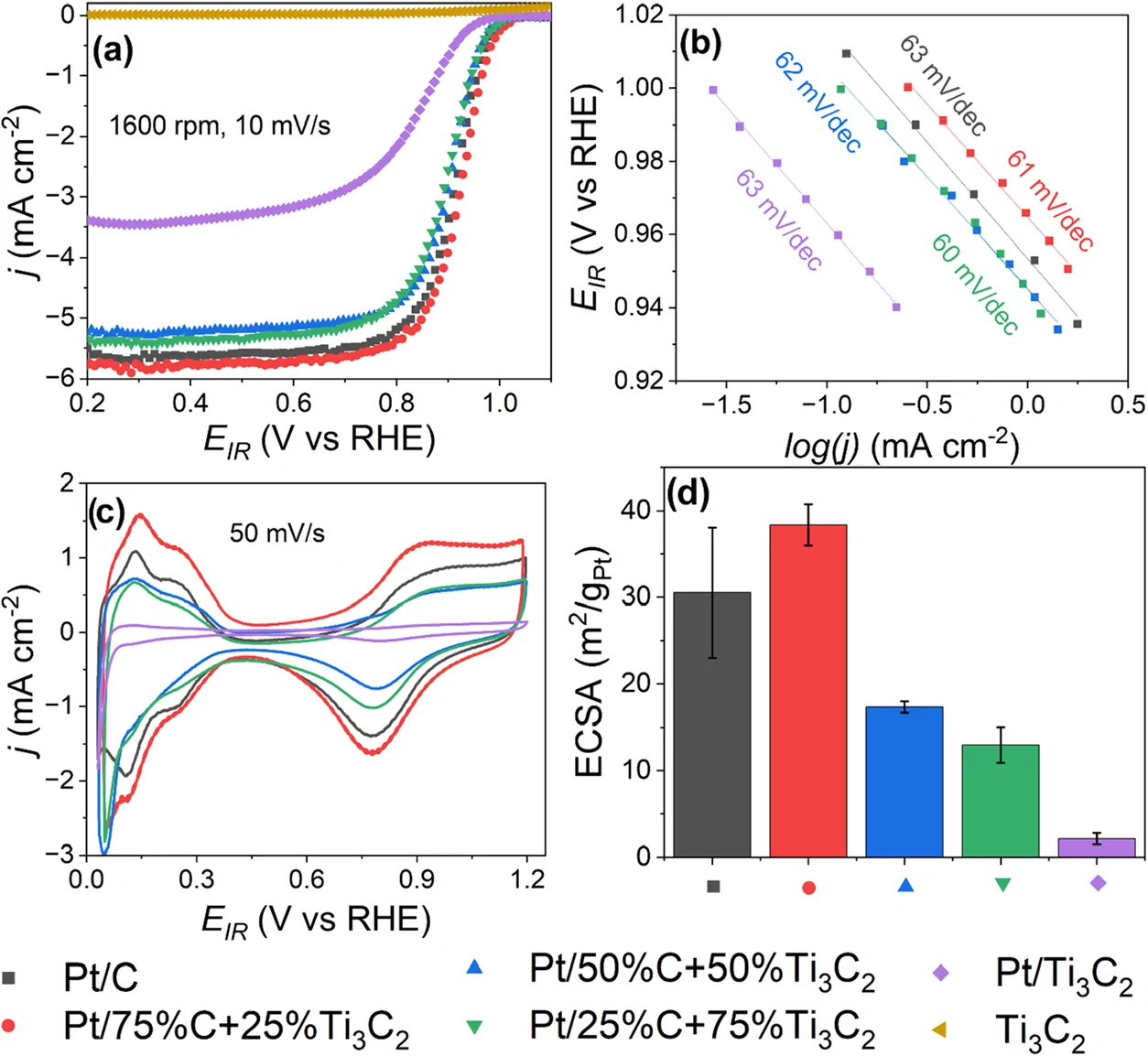
ORR RDE data in 0.1 M HClO_4_ at room temperature. (a) O_2_-saturated LSV at 1600 rpm, 10 mV/s. (b) Tafel plots from kinetic currents. (c) CV curves taken in N_2_-saturated electrolytes. (d) ECSA calculated from H_upd_.

All samples follow a 4-electron pathway for the reaction according to Koutecký–Levich analysis (Figure SI10), which indicates the reduction of O_2_ to water. Stability of the Pt/MXene and Pt/C+MXene samples was extensively studied in the literature using the RDE;^3,4,23,24^ however, there are no stability reports on the half-cell operations, which reflect the real working conditions much better than RDEs. Therefore, stability studies are performed in a Gaskatel flow cell.

Half-cell flow setups are important to investigate the realistic mass transport phenomena in the electrode and the catalyst layer. Figure 7a,b shows the half-cell performances for the ORR in 1.0 M HClO_4_ solution. Pt/75%C+25%Ti_3_C_2_ is the most active cathode, delivering 0.73 V at 1 A/cm^2^, followed by in-house-made and commercial Pt/C (0.71 V), Pt/50%C+50%Ti_3_C_2_ (0.66 V), Pt/25%C+75%Ti_3_C_2_ (0.2 V), and Pt/Ti_3_C_2_ (−0.57 V). The latter two samples are well out of the desired ORR range, especially Pt/Ti_3_C_2_, which could be attributed to the low mass transport caused by low surface area and low electrical conductivity caused by oxidized Ti species. All carbon-containing electrodes reveal similar onset potentials (∼0.95 V), while the Pt/Ti_3_C_2_ sample shows an inadequate onset potential (0.07 V). Rotating-disk measurements in 0.1 M HClO_4_ corroborate these trends: onset potentials cluster at ∼1.02 V (Pt/75%C+25%Ti_3_C_2_ highest at 1.03 V), limiting currents at 0.2 V, and all composites follow a 4-electron pathway. Tafel slopes (Figure SI11a) of the gas diffusion electrodes are 55, 56, and 59 mV/dec for Pt/75%C+25%Ti_3_C_2_, Pt/C, and Pt/50%C+50%Ti_3_C_2_, respectively. The Pt/25%C+75%Ti_3_C_2_ sample has a Tafel slope of 63 mV/dec, whereas Pt/Ti_3_C_2_ exhibits 168 mV/dec slope. The mass activities of the catalysts at 0.85 V were calculated as 0.120, A/mg, 0.133 A/mg, 0.047 A/mg, 0.032 A/mg, and 0.0001 A/mg for Pt/C, Pt/75%C+25%Ti_3_C_2_, Pt/50%C+50%Ti_3_C_2_, Pt/25%C+75%Ti_3_C_2_, and Pt/Ti_3_C_2_, respectively.

**Figure 7:**
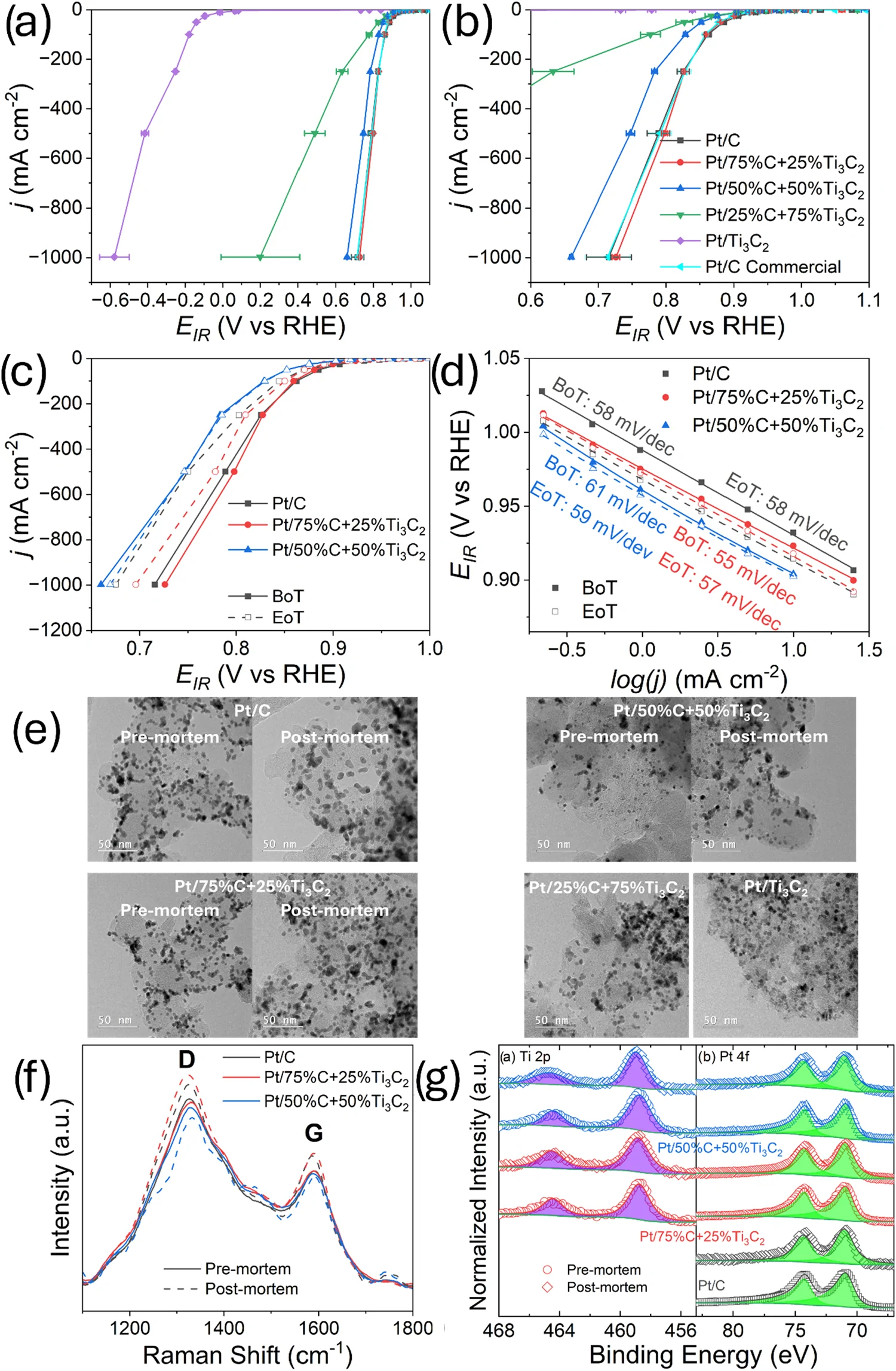
Gas-diffusion half-cell ORR in N_2_-saturated 1.0 M HClO_4_ at room temperature with O_2_ gas flow. (a) Polarization curves. (b) Zoomed-in polarization curves. (c) BoT vs EoT polarization in O_2_-fed half-cells after 30,000 potential cycles under N_2_. (d) Tafel slopes change minimally from BoT to EoT, indicating negligible kinetic alteration. (e) TEM micrographs and size histograms pre- and poststress: Pt/C shows the largest coarsening (mean: 5.3 nm pre, 7.3 nm post; > 10 nm from 4% to 19%), Pt/50%C+50%Ti_3_C_2_ is moderate (5.1 to 6.4 nm; > 10 nm from 2.4% to 10.8%), and Pt/75%C+25%Ti_3_C_2_ is least (5.2 to 5.9 nm; > 10 nm from 3.6% to 7.9%); premortem-only statistics are included for Pt/25%C+75%Ti_3_C_2_ (mean: 5.7 nm; 66% in 2–6 nm; 6.3% > 10 nm) and Pt/Ti_3_C_2_ (mean: 4.8 nm; 83% in 2–6 nm; 2.6% > 10 nm). (f) Premortem and postmortem Raman spectroscopy results of the half-cell electrodes showing the change in carbon peaks. (g) Ti 2p and Pt 4f spectra of electrodes before (BoT) and after (EoT) cycling; peak positions and component ratios are unchanged, indicating stable oxidation states and no compositional change. The presented performance curves in plots (a, b) are the mean values of three separate electrodes measured for each sample. Each electrode was measured only once. The error bars were calculated using the standard deviation for each point using the data from three separate electrodes.

Galvanostatic electrochemical impedance spectroscopy (GEIS) was used to determine the *iR* correction value, observing the behavior of the electrodes under load, given in Figure SI11b–d. GEIS at fixed current densities shows the lowest charge-transfer resistances for Pt/75%C+25%Ti_3_C_2_ at all loads. At −500 mA/cm^2^, Pt/25%C+75%Ti_3_C_2_ lacks a clear semicircle. At −1000 mA/cm^2^, both Pt/25%C+75%Ti_3_C_2_ and Pt/Ti_3_C_2_ are noisy and non-semicircular. The combined evolution of midfrequency arcs and low-frequency features is consistent with superior charge-transfer and transport characteristics for Pt/75%C+25%Ti_3_C_2_, followed by Pt/C and then Pt/50%C+50%Ti_3_C_2_ with severe transport limitations for the others. Charge transfer resistance values are given in Table 1. The comparison of the polarization curves with and without *iR* compensation is given in Figure SI12a.

**Table 1:** Charge Transfer Resistances of the Samples

	Charge transfer resistance (mΩ)				
Current Density (mA/cm^2^)	Pt/C	Pt/75%C+25%Ti_3_C_2_	Pt/50%C+50%Ti_3_C_2_	Pt/25%C+75%Ti_3_C_2_	Pt/Ti_3_C_2_
–250	370	290	460	1940	990
–500	230	190	320	–	530
–1000	140	120	200	–	–

Figure 7c,d shows the performances and Tafel slope results of the two best-performing MXene carbon composite electrocatalysts with the in-house-made Pt/C at the beginning of the test (BoT) and at the end of the test (EoT) after 30,000 cycles of potential cycling under an N_2_ flow for Pt degradation, from the DoE-derived stability protocol.^22^ Figure 7c reveals the performance change after cycling. At 1.0 A/cm^2^, Pt/50%C+50%Ti_3_C_2_ shows no performance loss, contrary to other samples which show 4% (30.2 mV for Pt/75%C+25%Ti_3_C_2_) and 6% (40.4 mV for Pt/C) performance losses. It is evident that the increasing Ti_3_C_2_ amount increases the stability. However, the lowest performance out of all three samples corresponds to Pt/50%C+50%Ti_3_C_2_. The highest performance belongs to Pt/75%C+25%Ti_3_C_2_, followed by Pt/C, which indicates a superior lifetime for the Pt/75%C+25%Ti_3_C_2_ sample. Figure 7d shows the change in the Tafel slope after the degradation test. The minimal change in the slopes combined with the performance results indicates a negligible change in the Pt performance.

To elucidate a possible rationale for the increased performance of the Pt/75%C+25%Ti_3_C_2_, postmortem analysis was carried out. SEM analysis on carbon cloth for premortem (before the stability test) and postmortem (after the stability test) was carried out and shows no significant change in the electrodes (Figure SI13). No cracks in the support or changes in morphology were observed. TEM pre- and postmortem analysis of the electrodes revealed the change of the average Pt nanoparticle size after the stress test, given in Figure 7e, and the histograms showing the Pt NP sizes and change are given in Figure SI14. TEM size histograms indicate coarsening after the stress test. For Pt/C, the mean particle size increased from 5.3 to 7.3 nm, and the fraction of particles larger than 10 nm rose from 4% to 19%. For the Pt/50%C+50%Ti_3_C_2_ sample, the mean size increased from 5.1 to 6.4 nm, with the fraction above 10 nm growing from 2.4% to 10.8%. The Pt/75%C+25%Ti_3_C_2_ sample changed the least, with the mean size increasing from 5.2 to 5.9 nm and the fraction above 10 nm rising from 3.6% to 7.9%. The share of particles in the 2–6 nm range dropped the most in Pt/C (27%), followed by Pt/50%C+50%Ti_3_C_2_ (13.6%) and Pt/75%C+25%Ti_3_C_2_ (7.8%). These results indicate that the mixed support with the higher carbon fraction, Pt/75%C+25%Ti_3_C_2_, provides the best resistance to particle growth during the stress test. For the Pt/25%C+75%Ti_3_C_2_ sample, the mean Pt particle size was 5.7 nm with a median of 5 nm, and the distribution peaked at 3 nm; about 66% of the particles lay between 2 and 6 nm, and ∼6.3% were larger than 10 nm. For the Pt/Ti_3_C_2_ sample, the mean size was 4.8 nm with a median of 4 nm and a mode of 3 nm; about 83% of the particles were between 2 and 6 nm, and ∼2.6% exceeded 10 nm.

Another important factor to decide after the AST is the chemical stability of the support for the Pt (e.g., pure carbon vs MXene and carbon) given in Raman spectra in Figure 7f. The accelerated stability conditions preferentially oxidize and remove defective/edge carbon. In Raman spectroscopy, the oxidation and removal of defective/edge carbon can be easily detected by changes in the D/G intensity ratio and, depending on the extent of damage to the surviving framework, by shifts and width changes of the G band. Raman spectra for pre- and postmortem analysis of the spray-coated carbon cloth electrodes revealed the expected D and G bands at ∼1335 cm^–1^ and ∼1584 cm^–1^, respectively; they were analyzed using local baseline subtraction to quantify the structural disorder after the stability tests. The D/G intensity ratio decreased by 11.2% for Pt/C, from 1.69 to 1.50. Also, the G band peak shifted from 1584.2 cm^–1^ to 1582.8 cm^–1^ and narrowed (fwhm from 92.1 to 81.8 cm^–1^). This drop in D/G together with the G downshift indicates the removal of highly defective/edge carbon during the test, while the G narrowing indicates that the surviving carbon is somewhat more ordered. This trend is consistent with the greater overall carbon loss under cycling, matching the larger performance loss and stronger Pt coarsening after the stability test. The D/G intensity ratio decreased by 0.6% for Pt/75%C+25%Ti_3_C_2_, from 1.61 to 1.60. Also, the G band remained centered at 1583.2 cm^–1^ with a small narrowing (fwhm from 92.8 to 87.1 cm^–1^). The nearly unchanged D/G together with a stable G position and only minor G narrowing indicates that the graphitic framework was largely preserved with minimal carbon restructuring.

This aligns with the smaller activity loss and the smallest Pt nanoparticle growth observed after the stability test, consistent with reduced Pt coarsening for this composition. The D/G intensity ratio slightly decreased by 7.2% for Pt/50%C+50%Ti_3_C_2_, from 1.62 to 1.51. The G band peak shifted from 1583.7 cm^–1^ to 1587.7 cm^–1^ and narrowed (fwhm from 95.3 to 80.1 cm^–1^). The small D/G decrease together with a G upshift and peak narrowing indicates reduced disorder and an improved structure after the stability test, which is consistent with a carbon framework that undergoes less structural change and maintains robust Pt anchoring. This spectral behavior is coherent with the excellent durability outcome for this sample. After testing, Pt/C exhibits the lowest final D/G and the most downshifted G band, indicating that defective/edge carbon was preferentially consumed and that this sample underwent the greatest overall carbon corrosion. The Pt/75%C+25%Ti_3_C_2_ composite shows an almost unchanged D/G (1.60) with a stable G band (∼1583 cm^–1^), reflecting preserved graphitic integrity. The Pt/50%C+50%Ti_3_C_2_ composite shows a D/G of 1.51 with the most upshifted G band (1587.7 cm^–1^) and the greatest G narrowing (to 80.1 cm^–1^), indicating a comparatively ordered, less-strained network. Because absolute D/G can be influenced by the carbon type and microtexture, the within-sample pre- and postmortem trends and the G-band position/width are the more reliable indicators of degradation here. These indicators rank the extent of carbon structural change as Pt/C > Pt/50%C+50%Ti_3_C_2_ ≈ Pt/75%C+25%Ti_3_C_2_, in agreement with the electrochemical durability results.

XPS analysis was conducted on the catalysts pre- and postmortem to assess surface changes. Figure 7g shows the oxidation state differences between BoT and EoT electrodes, where the oxidation states and atomic concentrations do not change for Ti 2p and Pt 4f. This indicates that there is no change in the composition of the materials. Metal dissolution into the electrolyte was quantified by ICP-OES after both accelerated stress tests, for Pt and for Ti, with the dissolved amounts normalized to the initial Pt loading and to the Ti_3_C_2_-derived support content, respectively (Figure SI15). ICP-OES measurements indicate low but quantifiable Ti release into the electrolyte during the accelerated stress test. Since Pt/25%C+75%Ti_3_C_2_ and Pt/Ti_3_C_2_ are inactive across the ORR potential window, they were therefore not subjected to the durability protocol and ICP-OES measurements. Under the Pt-cycling AST, Pt dissolution is low for all catalysts: 9.7, 19.5, and 20 μg of Pt per mg of Pt for Pt/C, Pt/75%C+25%Ti_3_C_2_, and Pt/50%C+50%Ti_3_C_2_, respectively, with the commercial Pt/C reference at 20.6 μg mg^–1^. The corresponding Ti release for the two composites is likewise small—6.42 and 4.07 μg of Ti per mg of Ti in the support (0.64% and 0.4% of the initial Ti) for Pt/75%C+25%Ti_3_C_2_ and Pt/50%C+50%Ti_3_C_2_, respectively, confirming that the support is only marginally attacked in this potential window. The close values indicate that the Ti dissolution is minimal and only weakly dependent on the carbon/support ratio under the applied conditions. The other postmortem results support these findings. Postmortem XPS shows no change in the Ti 2p state as mentioned before, and postmortem Raman spectroscopy indicates that the dominant structural change occurs in carbon. Taken together with the low-dissolved Ti detected by ICP-OES, these results indicate that the Ti support remains largely intact under this Pt-cycling protocol, consistent with degradation being dominated by Pt particle growth rather than support loss. The total Ti released during the accelerated stress test is small (1.6–2.0 μg per electrode), and multivalent-cation poisoning of perfluorosulfonic acid ionomers scales with the concentration of mobile, exchangeable cations, which for such sub-microgram quantities remains far below the level associated with measurable proton conductivity loss. Unlike strongly Fenton-active contaminants such as Fe^3+^, dissolved Ti^4+^ hydrolyzes readily in acidic media and tends to reprecipitate as insoluble hydrous TiO_2_ rather than persisting as a mobile cation that can ion-exchange into the ionomer, which limits its capacity to poison the membrane. This is consistent with our postmortem Ti L-edge XANES and Ti 2p XPS, which show the Ti^4+^ oxide phase to be invariant after cycling, indicating that any restructuring of the titania occurs in place and retains Ti on the electrode rather than releasing it. The contribution of dissolved Ti or TiO_2_ restructuring to ionomer or electrolyte contamination is expected to be minor under the tested conditions, although direct confirmation over extended operation would require long-term measurements in an MEA configuration. Residual fluoride and etchant species from MXene synthesis are expected to be minimal, as the Ti_3_C_2_ was washed to neutral pH prior to use and then oxidized into TiO_2_ during the catalyst synthesis; combined with the low absolute Ti release measured here, any dissolved Ti or fluoride interaction with the ionomer or electrolyte is expected to be negligible over the tested protocol. Taken together, the suppressed Pt dissolution and coarsening, the preserved carbon framework, and the invariant Ti^4+^ oxide identify the durability gain of the MXene-containing supports as a combination of Pt anchoring at the anatase surface and the superior corrosion resistance of that oxide, rather than an electronic metal–support interaction.^29,32^

Figure 8a,b shows the performance and Tafel slope results of the two best-performing MXene carbon composite electrocatalysts with the in-house-made Pt/C at the beginning of the test (BoT) and at the end of the test (EoT) after 30,000 cycles of potential cycling under an N_2_ flow for support degradation, from the DoE-derived stability protocol.^22^ Figure 8a reveals the performance change after cycling. Contrary to Pt degradation test results, at 1.0 A/cm^2^, Pt/50%C+50%Ti_3_C_2_ shows the highest performance loss with a 599 mV drop (from 0.66 to 0.06 V), while other samples show 291 mV (from 0.726 to 0.435 V for Pt/75%C+25%Ti_3_C_2_) and 330 mV (from 0.715 to 0.385 V for Pt/C) performance losses after the support degradation. The high drop rate of the Pt/50%C+50%Ti_3_C_2_ sample might stem from the low amount of carbon in the support. 25% Ti_3_C_2_ addition to the support causes the least amount of performance loss, which shows the improvement obtained by the Ti_3_C_2_-derived TiO_2_. The highest performance belongs to Pt/75%C+25%Ti_3_C_2_ after the support degradation test, followed by Pt/C, resulting in a superior lifetime for the Pt/75%C+25%Ti_3_C_2_. Figure SI12 shows the polarization curves with the middle of the test (MoT) results after 15,000 cycles. The first half of the test shows a small performance loss for Pt/C (36 mV) and Pt/75%C+25%Ti_3_C_2_ (46 mV) samples; however, the degradation is 155 mV for Pt/50%C+50%Ti_3_C_2_ even in the first half of the test. Figure 8b shows the change in the Tafel slope after the degradation test. The minimal change in Pt/C and Pt/75%C+25%Ti_3_C_2_ slopes combined with the performance results indicates a negligible change for the catalytic activity, while the Pt/50%C+50%Ti_3_C_2_ sample has a higher increase in the slope. SEM was used to examine the changes in the catalyst layer after the support degradation tests (Figure 8c). The white dots indicate the Pt NPs. The initially intact catalyst surface has become rough and defect-filled after the support degradation. The highest degradation is observed in the Pt/C with holes in the surface and the highest rate of coarsening of Pt NPs, as they can now be easily seen even at the 10 μm scale. Pt/50%C+50%Ti_3_C_2_ displayed the second worst degradation with a slightly less rough surface and Pt NP coarsening compared to Pt/C. Pt/75%C+25%Ti_3_C_2_ shows the most intact and smooth surface after the support degradation tests compared to the other two samples, with a lower Pt coarsening amount. Figure SI16 shows the EDX mapping of the postmortem electrodes. Pt NPs are uniformly distributed on the surface of the electrodes for all samples. The carbon signal slightly decreases with the increasing amount of MXene. The F signal comes from the carbon cloth itself, and the Cl signal is caused by the perchloric acid residue. A uniform Ti distribution can be observed in the C framework. Raman spectra of the pre- and postmortem electrodes after the support-degradation AST are shown in Figure 8d. All electrodes retained the characteristic D and G bands at ∼1335 cm^–1^ and ∼1584 cm^–1^. For Pt/C, the D/G intensity ratio decreased by 3.6%, from 1.69 to 1.63, with the G band shifting downward from 1584.2 cm^–1^ to 1582.7 cm^–1^ and narrowing (fwhm from 92.1 to 79.9 cm^–1^), consistent with the removal of a portion of the defective/edge carbon, while the surviving framework remained graphitic. For Pt/75%C+25%Ti_3_C_2_, the D/G ratio was nearly unchanged, from 1.61 to 1.58 (1.5% decrease), with a stable G position (from 1583.2 to 1583.5 cm^–1^) and only modest narrowing (fwhm from 92.8 to 84.3 cm^–1^), indicating that this composition best preserved its carbon framework under the support AST. In contrast, Pt/50%C+50%Ti_3_C_2_ showed the largest change, with the D/G ratio decreasing by 10.0%, from 1.62 to 1.46, a G downshift (1583.7 to 1582.4 cm^–1^), and essentially unchanged G width (95.3 to 93.1 cm^–1^), pointing to the most extensive carbon restructuring among the three. Because absolute D/G is influenced by the carbon type and microtexture, the within-sample pre/post-trends and G-band position/width are more reliable indicators here. These indicators rank the extent of carbon structural change under the support AST in the order of Pt/50%C+50%Ti_3_C_2_, followed by Pt/C, and finally Pt/75%C+25%Ti_3_C_2_, in agreement with the electrochemical durability results, where the Pt/50%C+50%Ti_3_C_2_ composition showed the largest high-current performance loss and the Pt/75%C+25%Ti_3_C_2_ composition showed the best retention.

**Figure 8:**
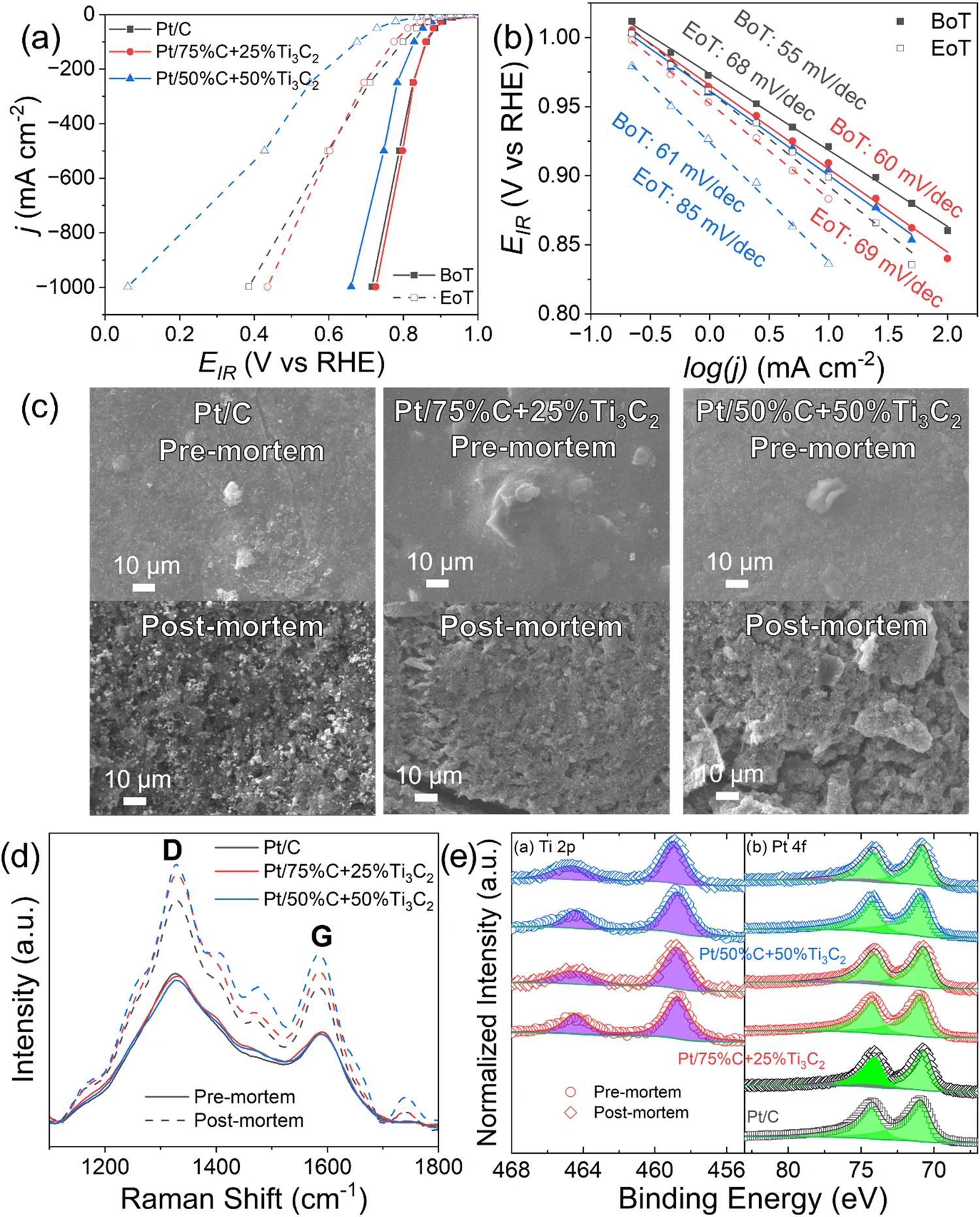
Accelerated support-degradation test in the gas-diffusion half-cell results. (a) Beginning-of-the-test (BoT) vs end-of-the-test (EoT) polarization curves. (b) Tafel slopes at BoT vs EoT. (c) SEM images of the catalyst layers after the support AST. (d) Pre- and postmortem Raman spectra. (e) Ti 2p and Pt 4f XPS spectra of the electrodes before and after the support-degradation AST.

Under the more aggressive support-corrosion AST (1.0–1.5 V), dissolution of both Pt and Ti increases substantially compared to the Pt AST, as expected for the higher potential protocol. Pt dissolution reaches 17.4% of the initial Pt loading for the in-house Pt/C, the highest of the series, whereas both composites lose considerably less, 10.3% for Pt/75%C+25%Ti_3_C_2_ and 13.2% for Pt/50%C+50%Ti_3_C_2_, and the commercial Pt/C loses 8.2%. The lower Pt loss of the MXene-containing catalysts relative to the in-house Pt/C, under precisely the conditions that drive support degradation, indicates that the Ti_3_C_2_-derived oxide helps retain Pt against dissolution. Ti release under this protocol is also higher—11.28 and 15.20 μg of Ti per mg of Ti in the support (1.128% and 1.520% of the initial Ti) for Pt/75%C+25%Ti_3_C_2_ and Pt/50%C+50%Ti_3_C_2_, respectively; the smaller fractional Ti loss of the 25% Ti_3_C_2_ composite indicates that the lower MXene support is the more chemically robust of the two. In both tests, the fraction of Ti lost remains below ∼1.5%, confirming that the oxide support is largely retained even under high-potential cycling, consistent with the invariant postmortem Ti 2p XPS and Ti L-edge spectra. The surface chemistry of the electrodes after the support-degradation AST was examined by XPS (Figure 8e). Despite the more oxidative 1.0–1.5 V window, the Ti 2p and Pt 4f core levels are essentially unchanged between the pre- and postmortem states for all catalysts. The Ti 2p region is described throughout by a single Ti^4+^ anatase doublet at 458.7–458.9 eV, with no Ti^3+^ or reduced/suboxide Ti component emerging after 30,000 cycles, indicating that the MXene-derived anatase support is neither further oxidized nor reduced under the support AST. Platinum remains fully metallic, with the Pt 4f_7/2_ and 4f_5/2_ peaks retained at 71.2 and 74.5 eV, respectively, and no growth of an oxidized Pt species. The atomic concentrations and Ti^4+^/Pt^0^ component ratios likewise remain constant within error, showing no measurable change in surface composition. Taken together with the invariant Ti L-edge multiplet, these results establish that the support and the Pt surface undergo no oxidation-state change even under the corrosive support-degradation protocol; the durability differences between compositions therefore arise from physical degradation, namely Pt crystallite growth and carbon-framework restructuring evidenced by postmortem XRD, SEM, and Raman, rather than from a chemical transformation of the support. The chemical inertness of the anatase component under these conditions is consistent with its role as a corrosion-resistant, stabilizing support rather than an electronically active participant in the degradation.

XRD data were collected on carbon cloth electrodes before and after 30,000 AST cycles, and the sharp 18° peak was chosen for normalization (Figure 9a for the Pt AST and Figure 9b for the support AST). The patterns are dominated by the carbon cloth and the sample holder below 30°; the catalyst-derived features are the face-centered cubic Pt reflections at 39.8° (111), 46.3° (200), 67.7° (220), and 81.4° (311), together with the carbon (002) band at ∼25.8°. All electrodes retained the fcc Pt structure after cycling. The principal postmortem change is a systematic narrowing and intensification of the Pt reflections in every electrode, indicating growth of the Pt crystallite size during potential cycling, and this narrowing is visibly more pronounced after the support AST (1.0–1.5 V) than after the Pt AST (0.6–0.95 V). The comparison between the Pt (111) peaks of Pt for pre- and postmortem electrodes reveals the size differences of the Pt NPs due to modest sharpening and narrowing of the peaks. Taller and narrower peaks for postmortem electrodes indicate Pt coarsening, even more so after the support AST than the Pt AST. In-house-made Pt/C develops the tallest and the narrowest Pt (111), while Pt/75%C+25%Ti_3_C_2_ and Pt/50%C+50%Ti_3_C_2_ show similar reflections, retaining markedly broader profiles. The MXene-containing supports therefore suppress Pt particle growth relative to carbon under both protocols, with the largest effect under the more corrosive support degradation cycles, where loss of the carbon support and the associated detachment and coalescence of Pt particles dominate the degradation of the in-house-made Pt/C benchmark.

**Figure 9:**
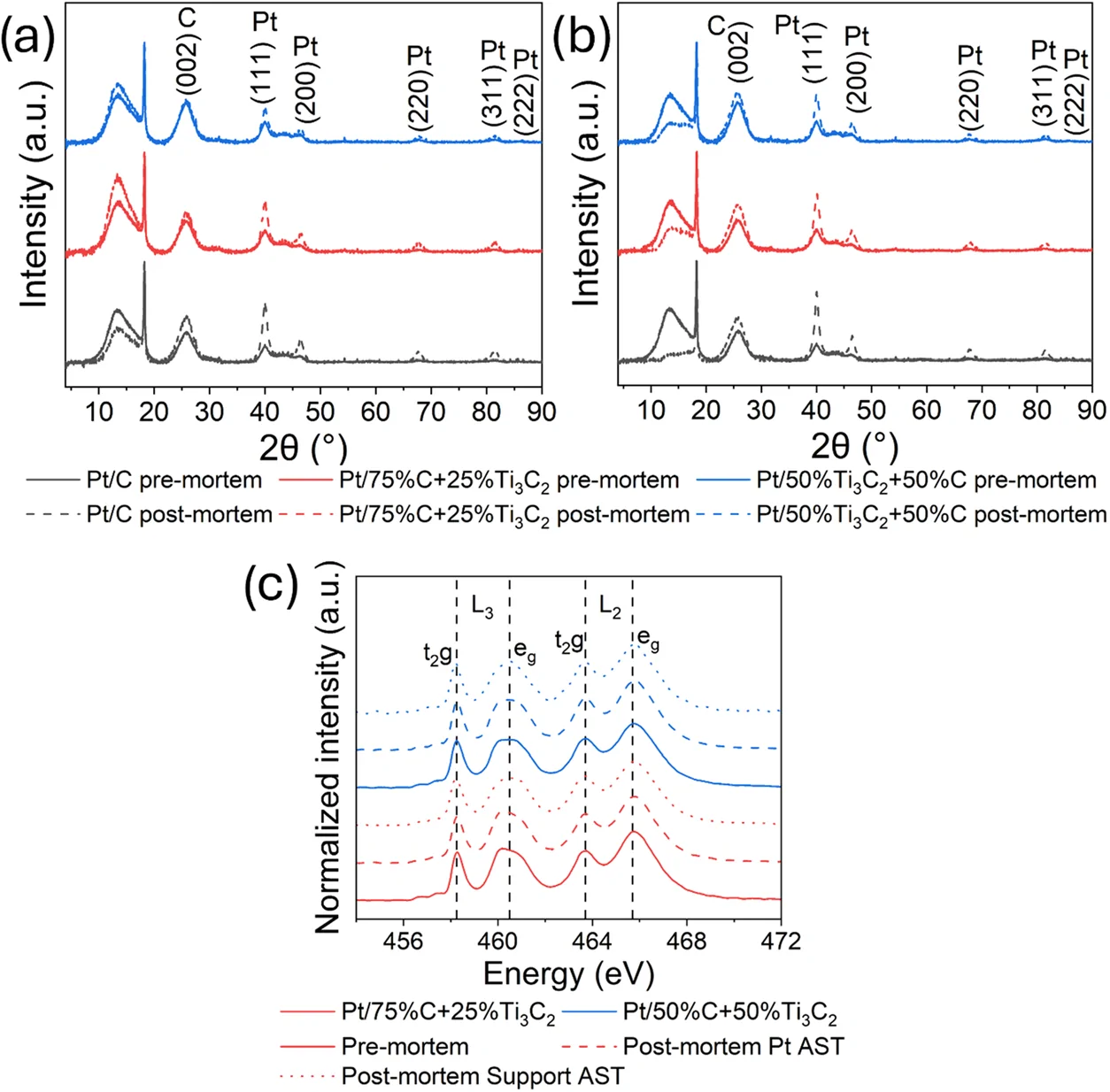
Postmortem XRD of the carbon cloth electrodes before and after 30,000 AST cycles. (a) Pt-AST (0.6–0.95 V) and (b) support-AST (1.0–1.5 V) patterns. (c) Ti L_2,3_-edge XAS of the Pt/75%C+25%Ti_3_C_2_ and Pt/50%C+50%Ti_3_C_2_ electrodes before and after both ASTs.

Ti L_2,3_-edge spectra were also recorded on the Pt/75%C+25%Ti_3_C_2_ and Pt/50%C+50%Ti_3_C_2_ electrodes before and after both accelerated stress tests, using the acquisition and normalization procedure applied to the powders (Figure 9d). In every case, the spectra retain the sharp Ti^4+^ anatase multiplet, with no emergence of reduced-Ti features and no loss of multiplet definition. Across all six electrode spectra, the L_3_ t_2_g position lies within 458.20–458.28 eV and the L_3_ e_g_ centroid lies within 460.50–460.55 eV, total spreads of 0.08 and 0.05 eV, respectively, comparable to the reproducibility of repeat scans of the same specimen. The e_g_/t_2_g ratio likewise shows no systematic change between the pre- and postmortem states of either composition. These observations are corroborated by Ti 2p XPS, which is described by a single Ti^4+^ doublet at 458.7–458.9 eV in every electrode, with no statistically justified Ti^3+^ component. The Ti speciation and local coordination of the support are therefore unchanged by 30,000 potential cycles under either protocol, including the 1.0–1.5 V support-degradation test. This is a direct measure of support-phase stability under the conditions that drive carbon corrosion, and it contrasts with the changes undergone by the other components of the same electrodes; postmortem XRD shows pronounced growth of the Pt crystallites, and Raman spectroscopy shows measurable modification of the carbon framework over the same tests. The MXene-derived anatase is thus not a participant in the degradation but a stable, corrosion-resistant component of the support, consistent with the reduced Pt coarsening and lower Pt dissolution measured for the MXene-containing catalysts. Table SI3 shows a literature comparison of this work and studies on other Ti_3_C_2_- and TiO_2_-based materials for the ORR in acidic media.

## Conclusions

This study provides the first AST protocol in a GDL flow cell with potential-cycling durability for Pt/C+Ti_3_C_2_ catalysts, enabling a direct benchmark against Pt/C in acidic media for the ORR. Incorporating both carbon and Ti_3_C_2_ MXene as the support for the active Pt allows simultaneous tuning of activity and durability; across all measurements, the carbon-rich composite (Pt/75%C+25%Ti_3_C_2_) shows the best overall balance. Structurally, XRD confirms fcc Pt on all samples, and SEM and TEM show thin Ti_3_C_2_ MXene flakes and uniformly distributed Pt nanoparticles, with ∼100 nm Pt clusters appearing more frequently in MXene-rich samples. HRTEM, STXM, XAS, and XPS reveal local anatase TiO_2_ on the Ti_3_C_2_ surfaces, and pre/postmortem XPS shows unchanged Pt 4f and Ti 2p states, indicating that degradation is dominated by particle growth rather than a change in the chemical state. The BET surface area decreases as the Ti_3_C_2_ amount increases due to restacking, except for the Pt/75%C+25%Ti_3_C_2_, which retains a larger surface area than that of Pt/C. RDE measurements show the highest ORR performance for Pt/75%C+25%Ti_3_C_2_ and Pt/C, with similar onset potentials and the strongest limiting currents. This is followed by Pt/50%C+50%Ti_3_C_2_ and then the Ti_3_C_2_-rich samples. The ECSA derived from RDE tests follows this trend. All Tafel slopes cluster near 60 mV/dec regardless of the support type, consistent with Pt kinetics, which shows that the RDE captures intrinsic kinetics but misses mass transport issues. GDL flow cell results confirm the hierarchy at the high-current regime: at 1 A/cm^2^, Pt/75%C+25%Ti_3_C_2_ delivers the highest voltage, followed by Pt/C and Pt/50%C+50%Ti_3_C_2_, while the Pt/25%C+75%Ti_3_C_2_ and Pt/Ti_3_C_2_ are well below the ORR range, showing the dominance of transport and kinetic limits. GEIS confirms that the lowest charge transfer/transport losses belong to Pt/75%C+25%Ti_3_C_2_, followed by Pt/C and then Pt/50%C+50%Ti_3_C_2_. The stability of the catalysts under the AST reveals complementary results. There is no voltage loss at 1 A/cm^2^ for the Pt/50%C+50%Ti_3_C_2_ sample, followed by only 4% loss for Pt/75%C+25%Ti_3_C_2_, which concludes that Ti_3_C_2_ addition increases the stability under this protocol, while Pt/C shows 6% loss after 30,000 cycles. However, the Pt/75%C+25%Ti_3_C_2_ sample still yields the best overall ORR performance after the AST and outperforms in-house-made Pt/C. TEM results show Pt coarsening for all samples, with the smallest growth observed for Pt/75%C+25%Ti_3_C_2_, followed by Pt/50%C+50%Ti_3_C_2_, and the largest growth for Pt/C. Raman D/G changes follow the same trend, which shows less carbon damage when MXene is present, but not dominant under this protocol. Under the more aggressive support-degradation AST (1.0–1.5 V), the compositional trade-off inverts. Pt/50%C+50%Ti_3_C_2_ suffers the largest high-current loss (599 mV at 1000 mA/cm^2^), whereas Pt/75%C+25%Ti_3_C_2_ retains the best performance, confirming that a carbon-rich composite is required to withstand the corrosive potential window and that 25% Ti_3_C_2_ is the practical optimum across both protocols. Postmortem XRD shows that the MXene-derived TiO_2_-containing supports suppress Pt crystallite growth under both tests, most strongly under the support AST, where Pt/C coarsens to 9.5 nm against 6.9 and 7.6 nm for the 25% and 50% Ti-containing composites, respectively. ICP-OES confirms lower Pt dissolution for the composites under the same protocol (10.3% for Pt/75%C+25%Ti_3_C_2_ versus 17.4% for the in-house Pt/C), while less than 1.5% of the support Ti is released, establishing that the anatase is largely retained and that degradation is dominated by Pt coarsening and carbon restructuring/loss. Pre- and postmortem Ti L_2,3_-edge XAS and Ti 2p XPS show the Ti^4+^ anatase phase to be invariant after 30,000 cycles under either protocol, with no emergence of reduced Ti, identifying the MXene-derived anatase as a stable, corrosion-resistant component rather than a participant in degradation. Consequently, a small amount of Ti_3_C_2_ improves the durability of the catalyst layer under the applied protocol, accompanied by suppressed Pt coarsening and, as indicated by postmortem Raman, less carbon structural change, while maintaining the electronic percolation and gas-transport pathways. This durability arises from a predominantly metal–support interaction, consistent with interfacial anchoring of Pt at the MXene-derived anatase surface through Pt–O–Ti bonds, together with the corrosion resistance of that oxide, and not from an electronic perturbation of Pt, which our Pt L_3_-edge XAS and Pt 4f XPS exclude even for the carbon-free composition. The excessive MXene amount lowers the ECSA, impairs mass transport, and diminishes activity. The Pt/75%C+25%Ti_3_C_2_ composition defines a practical window that maintains superior high-current performance after stress while avoiding the penalties of MXene-rich layers. By delivering the first coupled GDL flow cell performance-stability benchmark for Pt/C+Ti_3_C_2_, this study provides both an optimal composition target and a reproducible half-cell protocol for future Pt/MXene screening.

## Supplementary Material



## Data Availability

The data that support the findings of this study are available from the corresponding author upon reasonable request.

## Notes

Additional experimental details about materials and methods and results including SEM, TEM, STXM, XAS, BET, TGA, RDE, GDE, ICP-OES, and EDX (PDF)
