# Supplementary material for: Enhancing the Stability of Pt/Carbon with Ti3C2 MXene-Derived TiO2 for the Oxygen Reduction Reaction in Acidic Media
Source: J Am Chem Soc. 2026 Sep 28;148(39):42012–30. doi: 10.1021/jacs.6c09621 (PMC13651534; doi:10.1021/jacs.6c09621)
Supplement: Supplementary file 1 [file ja6c09621_si_001.pdf]

Supplementary Information

Enhancing the Stability of Pt/Carbon with Ti_3C_2 MXene-Derived TiO_2 for the Oxygen Reduction Reaction in Acidic Media

Mehmet Turan Görüryilmaz[a], Bastian Schmiedecke[a], Aline Alencar Emerenciano[a], Iris Dorbandt[a], Karuppasamy Dharmaraj[a], Thorsten Schultz[b,c], Xuyun* Guo[d]*, Bing Wu[e], Jiri Sturala[e], Namrata Sharma[f], Michael Haumann[g], Christian Göllner[h], Karl J. J. Mayrhofer[h], Zdenek Sofer[e], Norbert Koch[b,c], Valeria Nicolosi[d], Tristan Petit[f] and Michelle P. Browne[a]**

[a] Department of Catalytic Materials: Design and Integration, Helmholtz-Zentrum Berlin für Materialien und Energie GmbH, Hahn-Meitner-Platz 1, 14109 Berlin, Germany.

[b] Helmholtz-Zentrum Berlin für Materialien und Energie GmbH, 14109 Berlin, Germany.

[c] Institut für Physik & CSMB, Humboldt-Universität zu Berlin, 12489 Berlin, Germany.

[d] School of Chemistry, CRANN and AMBER Research Centers, Trinity College Dublin, Dublin 2, D02W9K7 Ireland.

[e] Department of Inorganic Chemistry, University of Chemistry and Technology Prague, 16628 Prague, Czech Republic.

[f] Helmholtz Young Investigator Group Nanoscale Solid-Liquid Interfaces, Helmholtz-Zentrum Berlin für Materialien und Energie GmbH, Hahn-Meitner-Platz 1, 14109 Berlin, Germany.

[g] Department Atomic-Scale Dynamics in Light-Energy Conversion (PS-ADLU), Helmholtz-Zentrum Berlin für Materialien und Energie GmbH Hahn-Meitner-Platz 1, 14109 Berlin, Germany.

[h] Forschungszentrum Jülich GmbH Helmholtz Institute Erlangen-Nürnberg for Renewable Energy (IET-2) Cauerstr. 1 91058, Erlangen, Germany.

***Corresponding Authors:** Mehmet Turan Görüryilmaz and Michelle P. Browne

E-mail: mehmet.goerueryilmaz@helmholtz-berlin.de, michelle.browne@helmholtz-berlin.de

Materials and Methods

Materials

Chloroplatinic acid hydrate ($H_2PtCl_6 \cdot xH_2O$, ≥ 99.9% trace metal basis, Sigma-Aldrich), ethylene glycol (EG, ReagentPlus®, ≥99%, Sigma-Aldrich), N,N-Dimethylformamide (DMF, 99.7+%, Thermo Scientific), carbon (Vulcan, Cabot Europa Ltd.), MAX Phase Ti_3AlC_2, (40 microns, Carbon-Ukraine), hydrochloric acid (HCl, 37%, ACS reagent, Sigma-Aldrich) and lithium fluoride (LiF, 300 mesh, Sigma-Aldrich), ethanol (99.8%, for HPLC, absolute, Thermo Scientific), isopropanol (99.5%, for HPLC, Thermo Scientific) perchloric acid ($HClO_4$, TraceSELECT™ Ultra, for ultratrace analysis, 67-72%), Nafion (5wt% solution, Quintech), DI water (Barnstead Smart2Pure Pro Water Purification System, Thermo Scientific) and nitrogen gas (Air Liquide, Alphagaz™ 1, 99.999% purity), carbon cloth with MPL+PTFE (CeTech W1S1011, thickness 410 µm), graphite rod (Alfa Aesar, 6.3 mm in diameter, 99%, metal basis), mini reversible hydrogen electrode (mini RHE, Gaskatel GmbH, Germany).

Methods

Ti_3C_2 MXene synthesis

Chemical etching with in-situ hydrofluoric acid (HF) method was used to synthesize the Ti_3C_2 MXene. 20 ml 9.0 M HCl was poured into a PTFE reactor and placed in an oil bath at 35 °C. Later 2.0 g of LiF was added slowly while being stirred with a magnetic bar for 30 minutes to form HF. After that, 1.0 g Ti_3AlC_2 was added slowly to the reactor and was stirred for 24 hours. After 24 hours, the solution was poured into 250 ml ultra-pure water to stop the reaction. The precipitate was washed with 250 ml ultra-pure water 3 times in a centrifuge at 3500 rpm for 5 minutes until it reached neutral pH. The black precipitate was diluted with 50 ml ultra-pure water and sonicated in a bath for 1 hour to obtain delaminated Ti_3C_2, followed by one more cycle of centrifugation for collecting delaminated nanosheets. Lastly, Ti_3C_2 was freeze dried and kept in a vacuum desiccator for further use.

Synthesis of catalysts

Pt electrocatalyst was synthesized using a solvothermal method by modifying the existing literature [1]. Appropriate amounts of carbon and/or Ti_3C_2 were weighed (40 mg C, 30 mg C+10 mg Ti_3C_2, 20 mg C+20 mg Ti_3C_2, 10 mg C+30 mg Ti_3C_2, 40 mg Ti_3C_2) in a beaker and 18 ml

DMF was added on top of the powder or powder mixture. Later it was sonicated in an ultrasonic bath for 20 minutes while being bubbled with N_2. Meanwhile, 0.082 g H_2PtCl_6 was dissolved in 12 ml of EG for 20 minutes while being bubbled with N_2. After 20 minutes, the EG solution containing Pt precursor was poured into the DMF solution containing the powder and further sonicated for 10 minutes while still being bubbled with N_2. The solvent system was chosen for two complementary roles. EG acts as a polyol solvent and mild reducing agent that reduces the Pt precursor to metallic Pt and limits particle growth, while DMF is a high-boiling polar aprotic co-solvent that disperses the carbon and Ti_3C_2 supports and additionally promotes Pt reduction and homogeneous nucleation on the support under solvothermal conditions. The solutions were transferred into a PTFE vessel and sealed in stainless steel containers. These containers were put into the oven at 180 °C for 8 hours and taken out of the oven when they naturally cooled down to room temperature. The solutions were poured into centrifuge tubes and washed 3 times with ethanol and then once with ultra-pure water, each for 10 minutes at 11 000 rpm. The samples were poured into petri dishes and left to dry at 80 °C overnight. Obtained electrocatalysts were named after their corresponding C/MXene ratios, such as Pt/C, Pt/75%C+25%Ti_3C_2, Pt/50%C+50%Ti_3C_2, Pt/25%C+75%Ti_3C_2, and Pt/Ti_3C_2. According to ICP-OES, the Pt loading on the carbon containing supports is 40%, while the loading on Pt/Ti_3C_2 is 36%.

Materials Characterization

The morphologies of the samples were examined by a field emission scanning electron microscope (FE-SEM) and the elemental mapping was conducted using energy dispersive X-ray spectroscopy (EDX), located at Correlative Microscopy and Spectroscopy (CCMS) Corelab (Helmholtz-Zentrum Berlin für Materialien und Energie, Germany) using a field emission FE-SEM instrument (Crossbeam 340 by Zeiss, Germany) along with a Bruker XFlash EDX system. The crystalline structure of the catalysts was determined by X-ray diffraction (XRD) at the X-ray Corelab at Helmholtz-Zentrum Berlin using a Bruker D8 instrument with a Cu Kα beam. The diffraction patterns were acquired using a step size of 0.02°. Raman spectra of the samples were collected for the samples with an i-Raman Plus spectrometer from BWTEK, using 785 nm laser excitation source at 10% laser power for powders and 15% laser power for the spray coated electrodes (maximum 340 mW at the probe exit). The Ti_3C_2 sample was a drop cast free standing film with 1 µm thickness, while the catalyst samples were prepared from the RDE ink, which were drop casted onto a microscope glass slide and dried at 80 °C on a hot

plate. The elemental composition and chemical state at the surface of catalyst samples were analyzed by X-ray photoelectron spectroscopy (XPS) at the ENERGIZE beamline at the Bessy II light source operated by Helmholtz-Zentrum Berlin. The materials were deposited via drop casting method on silicon wafer substrates for these measurements. An excitation energy of 800 eV and a pass energy of 100 eV were used for all XPS spectra shown here. A polycrystalline gold foil (Au 4f spectrum shown in **Figure SI 1**) was used for energy calibration (Au $4f_{7/2}$ = 84.00 eV) and resolution determination, yielding an experimental resolution of 0.45 eV. Sensitivity factors were calculated using the inelastic mean free path for elements by Seah and Dench [2], the photoionization cross sections after Yeh and Lindau [3] and the experimentally determined transmission function of the analyzer applied after Shirley background subtraction. Thermogravimetric analysis (TGA) was conducted to evaluate the thermal stability and compositional changes of the samples using a Setaram Themys system. The measurements were performed under an oxygen/argon atmosphere (1/4) with a constant flow rate of 50 mL/min. Prior to heating, the samples were equilibrated at 30 °C for 1 h to ensure thermal stabilization. Subsequently, the temperature was increased from 30 °C to 1050 °C at a heating rate of 10 °C/min. ICP-OES tests to determine the Pt loading were performed using Optima 8300 DV from PerkinElmer. The samples were prepared by dissolving 10 mg of powder in 10 ml aqua regia (3 h at room temperature, 2 h in 60 °C water bath, constant stirring), and then filtering the solutions through a 45 µm filter using a syringe followed by diluting the solutions 100 times with 2% HNO_3. X-ray absorption spectroscopy (XAS) at the Pt L_3-edge was conducted at beamline KMC-3 of the BESSY-II synchrotron (Helmholtz Zentrum Berlin, Germany). The experimental setup featured a Si[111] double-crystal monochromator, DXP-XMAP pulse-processing electronics (XIA) and a 13- element energy-resolving Si-drift detector (RaySpec) for X-ray fluorescence monitoring. Samples were mixed with boron nitride (BN) with a volumetric ratio of 1:5 (sample:BN) in a mortar and loaded into the holders. Energy axis of the monochromator calibration was achieved (accuracy ±0.1 eV) using a Pt foil. The beam had a spot size on the sample of 1.5 x 3.0 mm^2 determined by a focusing mirror and slits. The spectra were acquired using a continuous 5 scan mode of the monochromator, with each scan taking 5 minutes. 5 scans were averaged per sample. The XAS data were processed (dead-time correction, background subtraction, normalization) resulting in normalized XANES and EXAFS spectra. The k^3-weighted EXAFS spectra were simulated using in-house software and phase functions derived from the FEFF9 code. XAS for Ti L-edges were conducted at the

ENERGIZE beamline at the Bessy II light source operated by Helmholtz-Zentrum Berlin. The samples (both powder and catalyst coated carbon cloth electrodes) were loaded onto sample holders via carbon tape. Energy calibration was done on a gold foil. The post-processing of the data was conducted using Athena software.

For pre-mortem and post-mortem analyses of the gas diffusion electrodes, XPS was used to determine the chemical state at the surface of the catalyst using a JEOL JPS-9030 instrument with a base pressure of 2 × 10−9 mbar. The catalyst coated carbon cloth substrates were mounted on a sample holder via screws. Al-K_α radiation of a non-monochromated source was used for excitation with a power of 300 W. A hemispherical analyzer with a pass energy of 50 eV for the survey spectra and 20 eV for the narrow scans was used to detect the kinetic energy of the emitted electrons under normal emission, yielding an overall energy resolution of 1.1 eV. The binding energy scale of the analyzer was calibrated using argon sputter-cleaned polycrystalline gold and copper foils and setting the binding energies of the $Au4f_{7/2}$ and the $Cu2p_{3/2}$ peaks to 84.00 and 932.66 eV, respectively. For peak fitting, CasaXPS was used, employing Shirley backgrounds, asymmetric line shapes (LF(0.78,1.5,20,40)) for the metallic Pt4f peaks and symmetric line shapes (GL(30)) for the Ti2p. Nitrogen adsorption-desorption measurements were performed at 77 K using a NOVAtouch 2200 analyzer (Quantachrome Instruments, USA). The Brunauer-Emmett-Teller (BET) method was applied to determine the specific surface area, while pore size distributions were derived from the adsorption isotherms using the DFT porosity model. Transmission electron microscopy (TEM) and annular dark field scanning TEM (HAADF-STEM) were performed using an uncorrected FEI Titan with Schottky field emission S-FEG source operated at 300 kV. The microscope was equipped with both Bruker XFlash 6T-30 EDX and Gatan Quantum electron energy-loss spectroscopy (EELS) detectors for elemental analysis. The EELS spectrometer was operated with an energy dispersion of 0.5 eV per channel. Scanning Transmission X-ray Microscopy (STXM) measurements were performed in transmission mode at the high-vacuum (10^{-8} mbar) MYSTIIC endstation, of the UE-48 undulator beamline at the synchrotron Bessy II, operated by Helmholtz-Zentrum Berlin. STXM was employed in recent STXM studies of Ti_3C_2 MXene [4], [5]. X-ray absorption spectra were acquired at the Ti L-edge (450 – 475 eV) and O K-edge (528 – 550 eV) by raster-scanning the sample through the focused X-ray beam and recording the transmitted photon flux using an avalanche photodiode. Transmission data were converted to optical density (OD = $-\log(I/I_0)$), using Mantis software, where I is the transmitted

intensity through the sample and I_0 is the incident flux measured on a clean region of the SiN_x membrane. Catalyst inks were prepared by dispersing the powder samples (Ti_3C_2, and Pt/75%C+25%Ti_3C_2) in a H_2O/IPA mixture to a concentration of 10 mg/ml, with the addition of 8 µL Nafion solution to improve film adhesion. The inks were sonicated for 10 minutes in an ultrasonic bath to ensure homogeneous dispersion and subsequently diluted by dissolving 4 µL of the ink in 1 ml H_2O/IPA. Silicon nitride (SiN_x) membranes (Silson) with dimensions of 1 × 1 mm^2 (100 nm thickness) and supported on a 5 × 5 mm^2 Si frame (200 µm thickness) were used as substrates. A volume of 4.0 µL of the diluted ink was drop cast onto the SiN_x windows and allowed to dry at room temperature. Optical microscope images were recorded prior to measurement to identify regions of interest, and the windows were subsequently mounted onto the STXM sample holder. Dissolved Ti and Pt species released after the AST were quantified by ICP-OES using a Thermo iCAP 7400 ICP-OES spectrometer (Thermo Fisher Scientific) operated in axial view mode. The electrolyte solutions collected after the AST were analyzed directly, with Ti and Pt concentrations determined using the Ti 337.280 nm and Pt 265.945 nm emission lines respectively.

Electrochemical Characterizations

The intrinsic activities of the electrocatalysts were assessed in a rotating disc electrode system. 5.0 mg of each electrocatalyst powder was weighed and sonicated for 30 minutes in an ink made of 475 µl ethanol/DI Water (1:1) mixture and 25 µl 5% Nafion solution. Later, a 1.5 µl aliquot was drop cast onto a 0.07 cm^2 glassy carbon disk and rotated at 700 rpm for 30 minutes to dry. Glassy carbon was used as the working electrode. The counter electrode was a graphite rod, and the reference electrode was an RHE. 0.1 M $HClO_4$ was used as electrolyte. The electrolyte was bubbled with N_2 for 30 minutes prior to the catalyst layer activation and measurements were performed under an N_2 blanket. Consecutively, LSV at various rotating speeds and PEIS measurements were conducted in a fresh solution that was bubbled with O_2 for 30 minutes prior to the measurements and kept bubbling throughout the process. The background data taken in nitrogen-saturated solutions were subtracted from the oxygen-bubbled data to obtain the ORR results. For each catalyst, ink was deposited on three different glassy carbon electrodes and measurements were carried out on all of them.

GDL Half-Cell Measurements

Half-cell measurements were conducted to investigate the behavior of the catalysts in a realistic mass transport environment. The samples were spray coated with an airbrush onto separate carbon cloths (3 x 5.5 cm^2 strips) that were washed with ethanol in an ultrasonic bath for 15 minutes and dried overnight at 80 °C. The carbon cloth was placed in a petri dish with a mask on top that has a circular hole with a 1 cm diameter. The petri dish was placed onto a hot plate at 95 °C. Nitrogen gas at 1 bar was used to spray the catalyst inks. The electrodes were left to dry at 80 °C overnight. The catalyst ink contained IPA/DI Water (20:80) mixture and the catalyst to ionomer ratio was kept at 70:30 and the total powder amount was 1wt% in the solution [6]. Each composition was spray-coated and tested as 3 independent electrodes; Pt loading was 1.1 ± 0.1 mg/cm^2, iR correction was applied using the high-frequency resistance from EIS at each current density (100% post-correction; **Table SI 1**).

Gaskatel half-cell setup was used to test the ORR activity of the electrodes. 25 ml 1.0 M $HClO_4$ was used as electrolyte. The solution was bubbled with N_2 for 30 minutes prior to the testing and kept being bubbled throughout the measurements with a 50 ml/min flow rate. The prepared electrodes were used as working electrodes, PtIr wire was the counter electrode, and the reference electrode was RHE. The electrodes were first activated in 200 ml/min N_2 flow, then the gas flow was switched to 200 ml/min O_2 flow for the ORR measurements. The test protocol was shared by Ehelebe et al. [6] and slightly modified for our purposes, which is given in **Table SI 1**.

After the performance tests, accelerated stress tests (AST) were conducted on the electrodes to assess the durability of the catalyst layer. The protocol was adapted from U.S. Department of Energy (DoE) [7] and applied to half-cell setups in the literature [8]. For the Pt degradation tests, the performance test protocol was followed first, then the electrodes were cycled at 0.95 V and 0.6 V, 3 seconds each, for 30 000 cycles under N_2 flow at 200 ml/min to simulate ~5000 working hours to assess the catalyst-layer ageing [9]. After the cycling, another performance test protocol was conducted to determine the degradation of the catalyst coated electrodes. For the support degradation tests, the same protocol was followed with a difference of applied voltage, the samples were cycled between 1.0 - 1.5 V and performance test protocols were conducted after 15 000 and 30 000 cycles. All ASTs were done at room temperature.

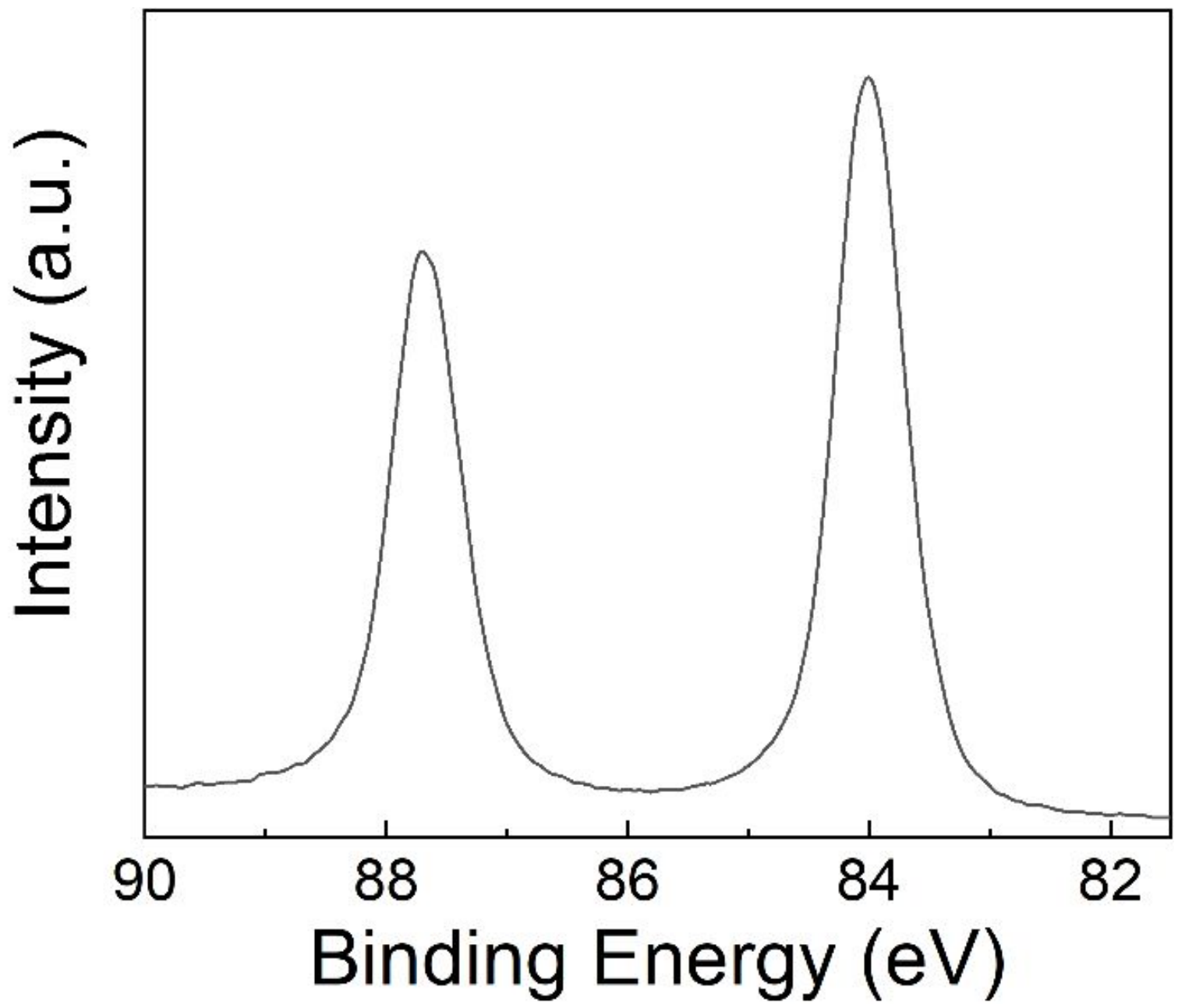

Figure SI 1: *Polycrystalline gold foil (Au 4f) spectrum used for energy calibration (Au 4f$_{7/2}$ = 84.00 eV) and resolution determination.*

***Table SI 1**: Half-cell testing protocol used for this study.*

	Step	Electrochemical Technique		Parameters
1	Start	Open circuit potential (OCP)	Gas purge (Flow rate)<br>Time	N_2 (200 ml/min), dry<br>10 min
2	Electrochemical Cleaning	Cyclic voltammetry (CV)	Gas purge (Flow rate)<br>Potential Limits<br>Scan rate<br>Number of cycles	N_2 (200 ml/min), dry<br>0.05 - 1.2 V vs RHE<br>200 mV/s<br>30 cycles
3	ECSA Determination (H_{upd})	CV	Gas purge (Flow rate)<br>Potential Limits<br>Scan rate<br>Number of cycles<br>iR-compensation	N_2 (200 ml/min), dry<br>0.05 - 1.2 V vs RHE<br>200-100-50 mV/s<br>3 cycles each<br>100% post-correction
4	Polarization Curve (O_2)	Open circuit potential (OCP)	Gas purge (Flow rate)<br>Time	O_2 (200 ml/min), dry<br>10 min
		Galvanostatic steps coupled with impedance spectroscopy (GEIS)	Gas purge (Flow rate)<br>Current steps (hold time)<br><br><br><br><br><br>EIS frequency range<br>EIS amplitude<br><br>iR-compensation	O_2 (200 ml/min), dry<br>-0.05/-0.1 mA/cm^2 (90s),<br>-0.25/-0.5 mA/cm^2 (60s)<br>-1/-2.5/-5/-10 mA/cm^2 (30s)<br>-25/-50/-100/-250 mA/cm^2 (5s)<br>-500/-1000 mA/cm^2 (5s)<br>f = 200 kHz - 10 Hz<br>10% of current (min 5 mA/cm^2)<br>100% post-correction

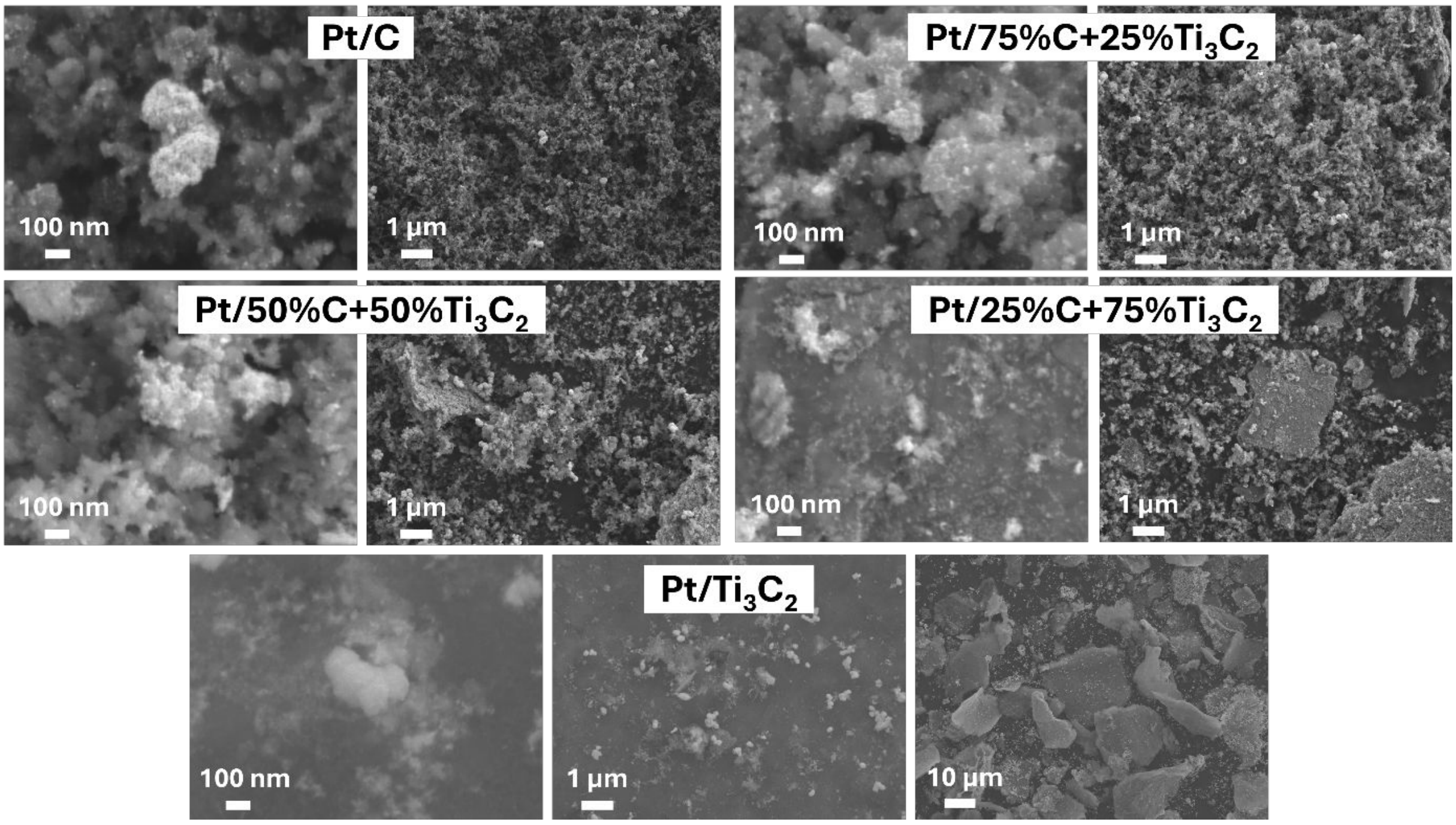

Figure SI 2: *SEM of powder catalysts: small carbon particles, large and thin Ti_3C_2 flakes and broadly dispersed Pt; occasional ~100 nm Pt clusters.*

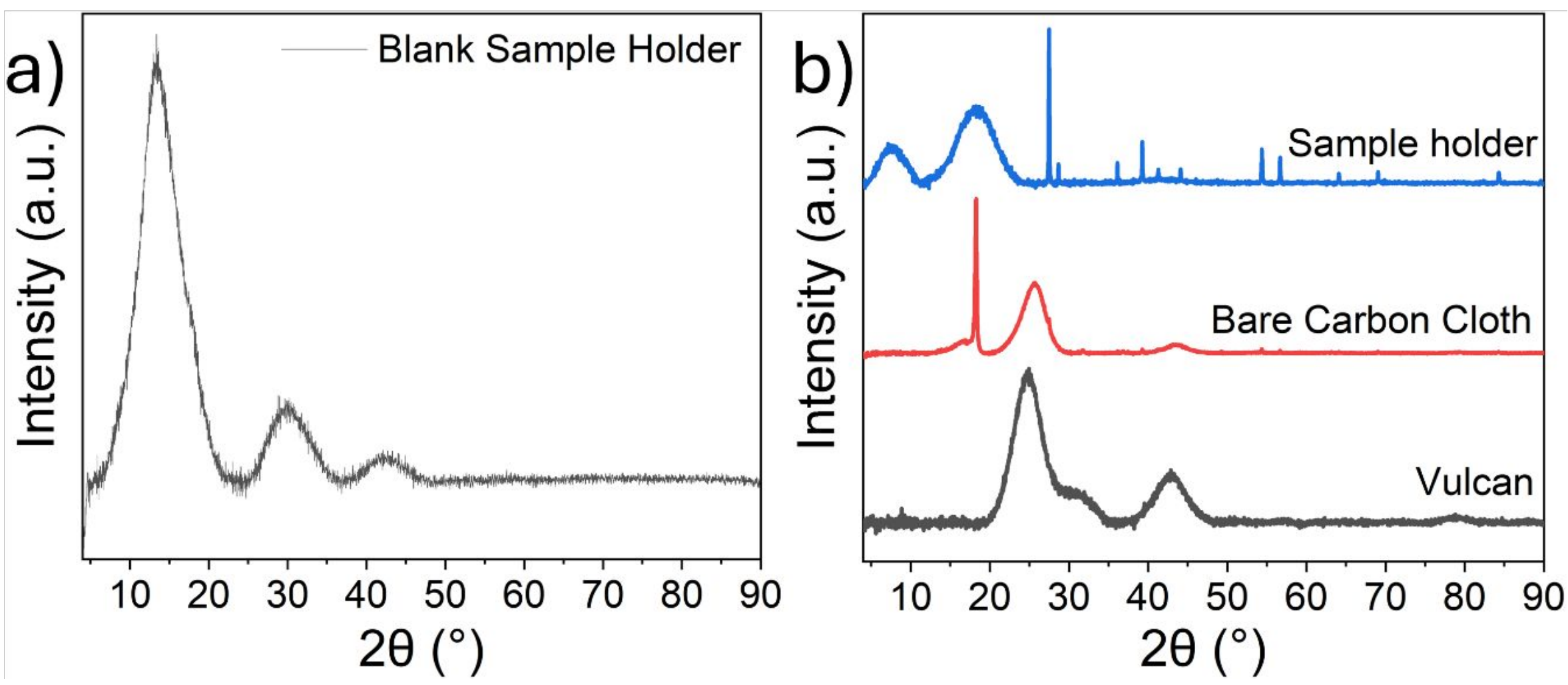

Figure SI 3: *a) XRD spectra of the blank sample holder for powders. b) XRD spectra of the sample holder used for catalyst coated carbon cloth electrodes, bare carbon cloth and Vulcan powder.*

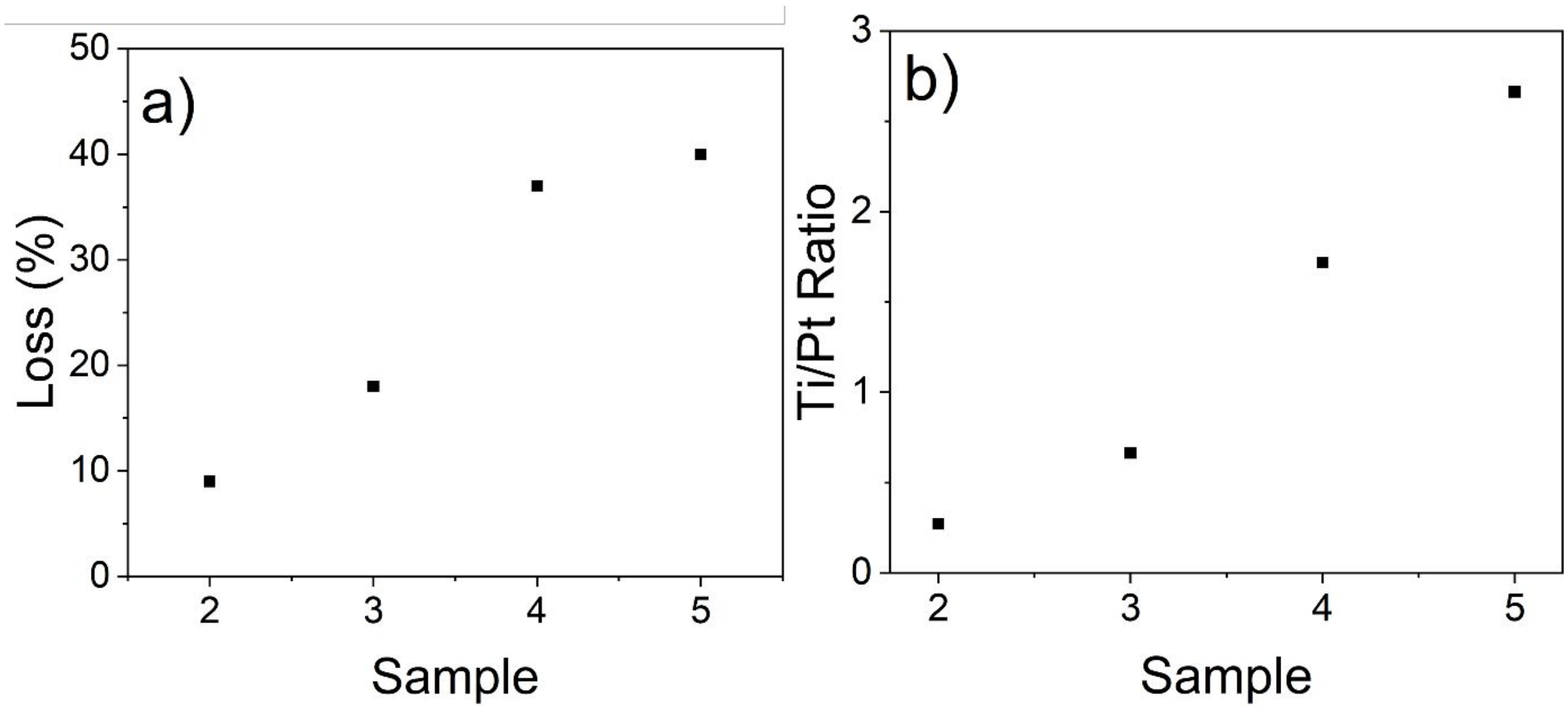

Figure SI 4: *The large-scale insets from Figure 5, a) Energy loss plot from Figure 4a(iii), b) Ti/Pt ratio from Figure 4a(ii).*

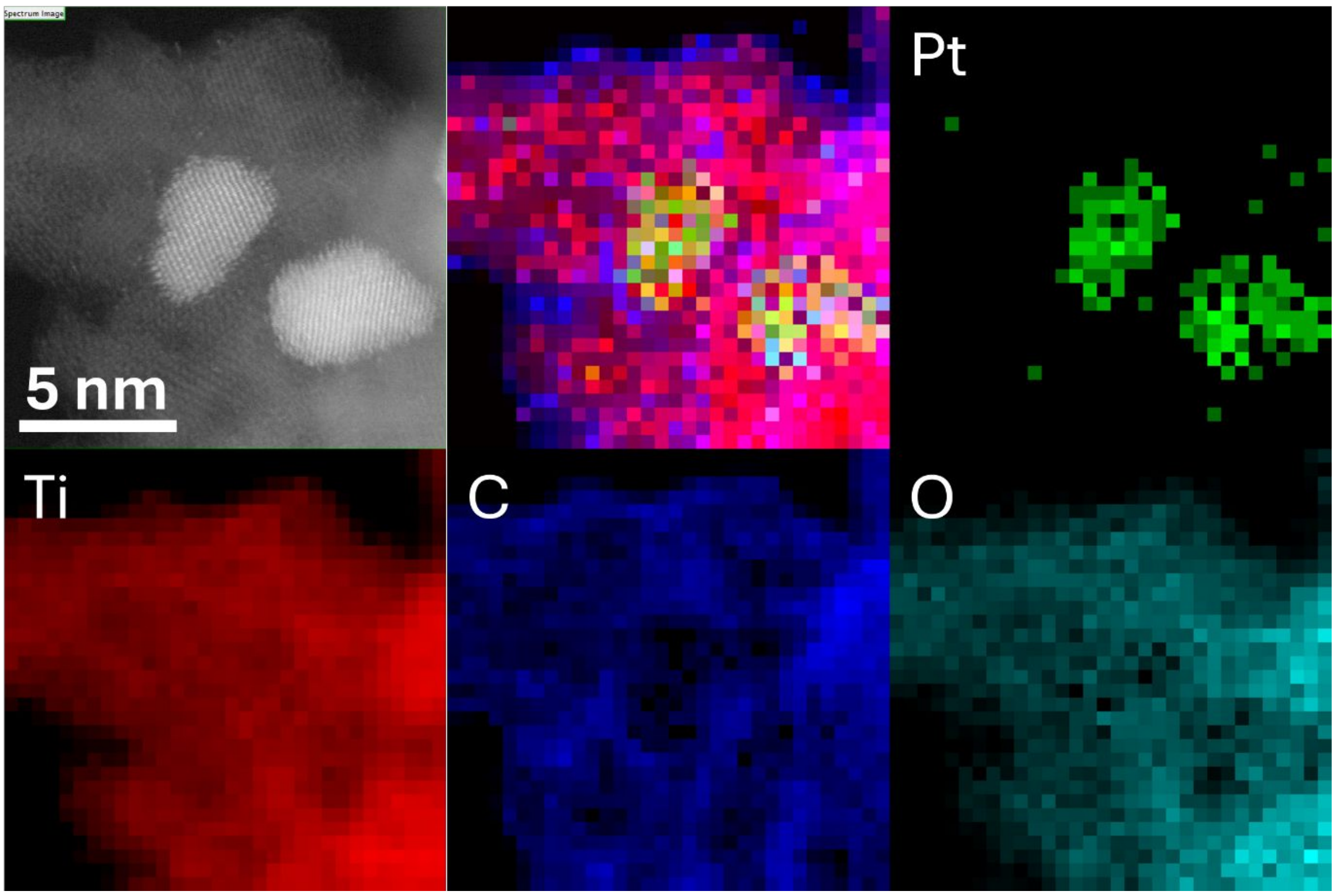

Figure SI 5: *High resolution MAADF-STEM image of Pt NPs anchored on TiO_2 support.*

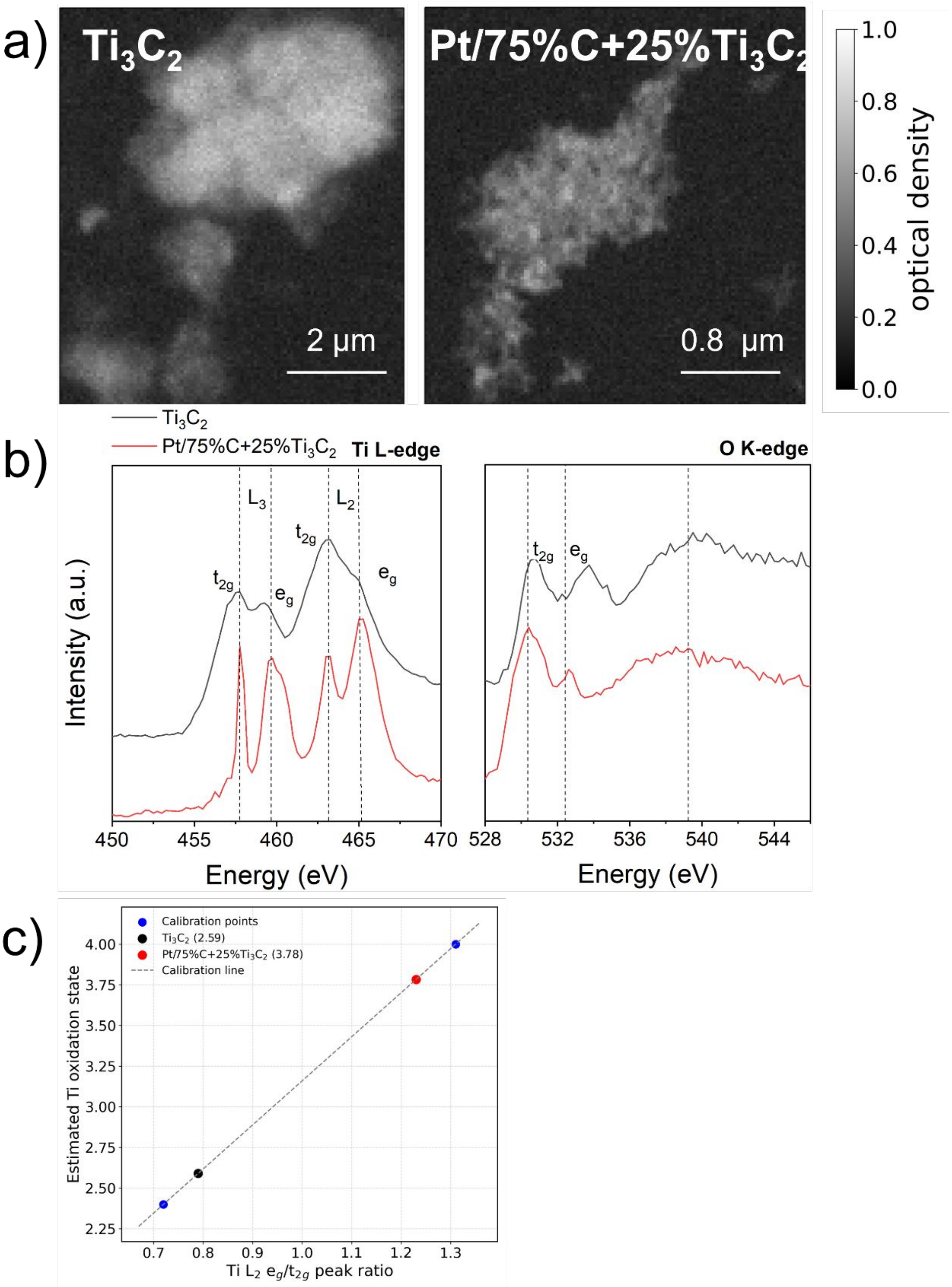

Figure SI 6: *a) STXM images of Ti_3C_2 MXene (black), and Pt/75%C+25%Ti_3C_2 (red), acquired at 463 eV with background in black and flakes/particles in white. b) Average XAS spectra of Ti L-edge (right) and O K-edge over flakes and particles. c) Ti oxidation state estimation.* ***.*** *Reference values of pristine MXenes and TiO_2 are taken from the work of Sharma et. Al* [5]. *The peak intensity values of L_2 t_{2g} and e_g peaks from main Figure 4c are used to estimate the Ti oxidation state of Ti_3C_2 (black) and Pt/75%C+25%Ti_3C_2 (red).*

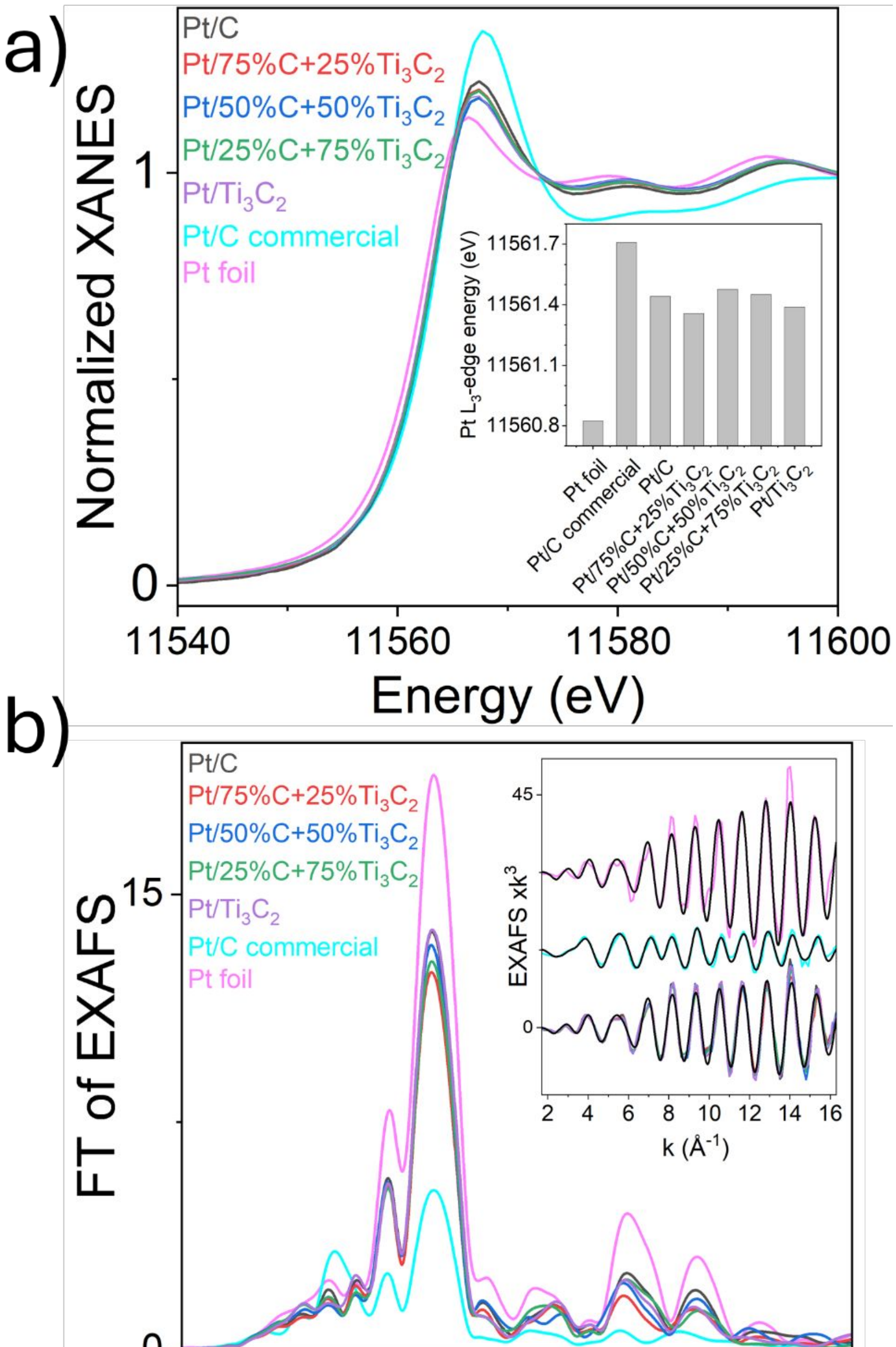

Figure SI 7: *Pt L_3-edge XAS spectra. Spectra were collected at room temperature (~22 °C). (A) XANES spectra of indicated samples and L_3-edge energies in the inset (determined by the integral method in a 0.15-0.9 XANES amplitude range). (B) Fourier-transforms (FTs, experimental data) of EXAFS spectra in the inset (colored lines, experimental data; black lines, simulations with parameters in Table SI 2). FTs were calculated for a k-range of 1.6-16.4 Å^{-1} using cos-windows over 10% at both range ends.*

Table SI 2. Pt EXAFS simulation parameters

(#coupled parameter; coordination number N [per Pt atom]; interatomic distance R [Å]; Debye-Waller factor $2s^2$ x10^3 [$Å^2$]; error sum R_F 1-3 Å [%].

#	Pt-O	Pt-Pt	Pt-Pt	R_F
**Pt foil**				
N	0.3	1.3	10.1	
R	2.10	2.51	2.77	15.8
$2s^2$	2	10#	10#	
**Pt/C commercial**				
N	1.9	1.7	2.2	
R	2.00	2.69	2.78	14.5
$2s^2$	11	8#	8#	
**Pt/C+Ti_3C_2 (mean)**				
N	0.6	0.6	8.4	
R	2.09	2.50	2.76	13.1
$2s^2$	3	11#	11#	

Figure SI 8 shows the adsorption/desorption curves of the samples. BET surface area results follow the expected trend of decreasing surface area with the increasing MXene content, except for Pt/75%C+25%Ti_3C_2. The surface areas of pure carbon and pure Ti_3C_2 are 160.2 m^2/g and 47.5 m^2/g, respectively. For the MXene carbon composites, the highest surface area can be attributed to Pt/75%C+25%Ti_3C_2 with 95.8 m^2/g, followed by Pt/C with 89.2 m^2/g, Pt/50%C+50%Ti_3C_2 with 67.5 m^2/g, Pt/25%C+75%Ti_3C_2 with 43.0 m^2/g and finally Pt/Ti_3C_2 with 35.7 m^2/g. The decrease of the surface area between pure carbon and Pt/C reflects the Pt filling and surface coverage. It is also seen that a small amount of Ti_3C_2 helps carbon to disperse better and create more surface area.

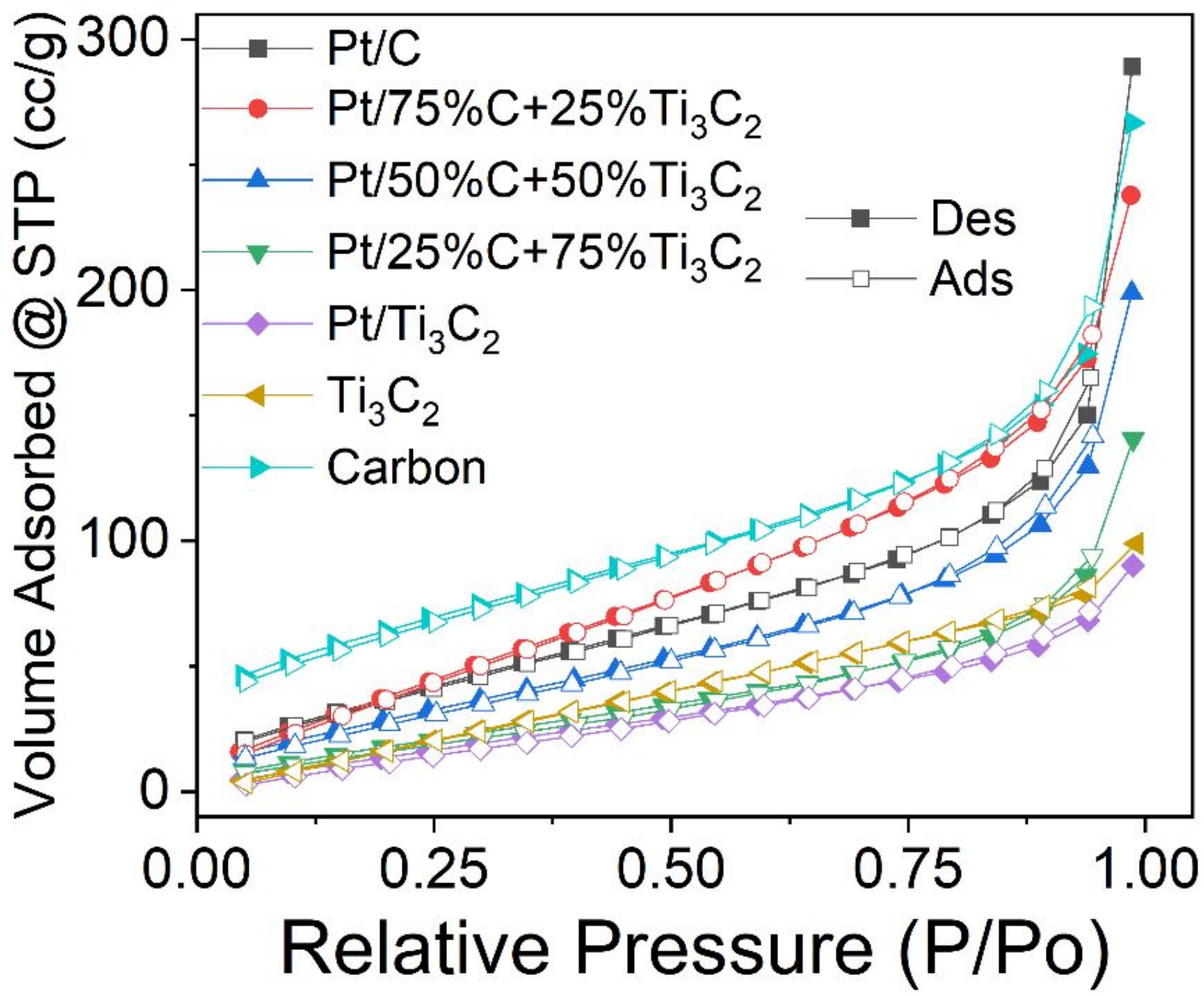

Figure SI 8: *Nitrogen adsorption–desorption isotherms at 77 K for the samples. Adsorption uptake decreases with increasing Ti_3C_2 fraction, consistent with BET areas. Pt deposition lowers uptake relative to parent supports, consistent with partial pore coverage/restacking.*

The thermal behavior of the samples was examined by TGA under an oxidizing O_2 atmosphere (**Figure SI 9**). All samples show a minor mass loss below 150 °C (0.7–1.6%) attributable to desorption of physisorbed water, which increases slightly with Ti_3C_2 content owing to the hydrophilic, hydroxyl-terminated MXene-derived surface. The principal mass loss occurs between 400 and 550 °C and corresponds to combustion of the carbon support. For the reference Vulcan carbon this combustion peaks at 686 °C, whereas for Pt/C the same process peaks at only 457 °C; the ~230 °C reduction reflects catalysis of carbon oxidation by the dispersed Pt nanoparticles. Introducing Ti_3C_2-derived oxide progressively shifts the carbon-combustion maximum back to higher temperature (484, 510 and 526 °C for Pt/75%C+25%Ti_3C_2, Pt/50%C+50%Ti_3C_2 and Pt/25%C+75%Ti_3C_2, respectively), indicating that the oxide component raises the onset of carbon oxidation and thereby enhances the thermal-oxidative stability of the support in proportion to its loading. The residual mass at 1000 °C, comprising metallic Pt together with the TiO_2 derived from oxidation of the MXene, increases systematically with Ti_3C_2 fraction (37.3, 57.2, 59.9, 75.6 and 85.0% for Pt/C, Pt/75%C+25%Ti_3C_2, Pt/50%C+50%Ti_3C_2, Pt/25%C+75%Ti_3C_2 and Pt/Ti_3C_2, respectively). The freeze-dried Ti_3C_2 reference shows a net mass gain (residue 104.7%), the characteristic signature of conversion of the carbide to TiO_2, in which uptake of oxygen outweighs the loss of carbon; this corroborates the oxidation of the MXene to titania inferred from XRD, XPS and the Ti L-edge and O K-edge spectra. Together, the TGA data confirm both the compositional trend across the series and the enhanced thermal-oxidative stability of the carbon support imparted by the MXene-derived oxide.

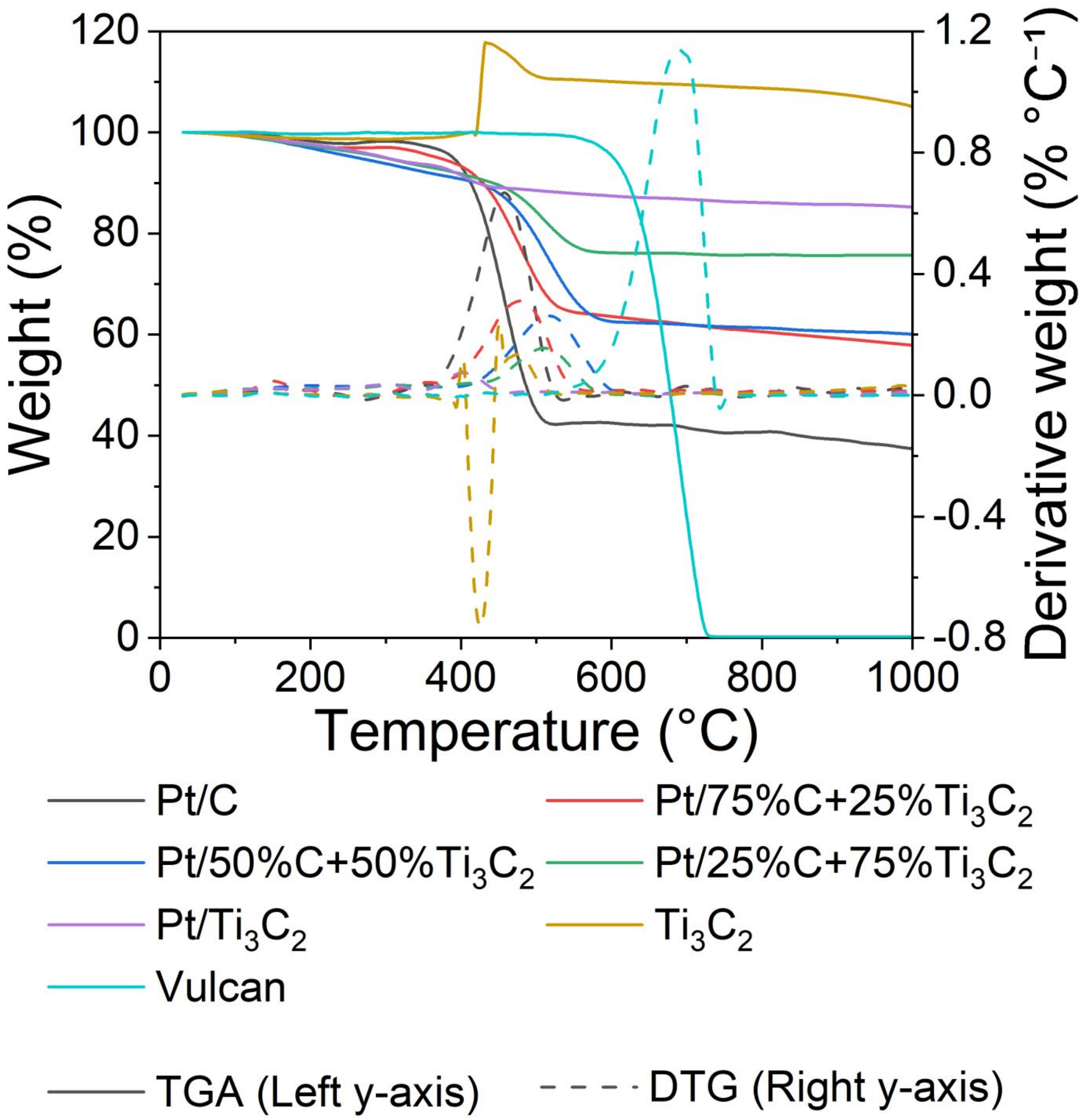

Figure SI 9: *Thermogravimetric analysis and derivative weight profiles of the samples measured in O_2/Ar up to 1000 °C. An initial low-temperature mass decrease is attributed to desorption of physically adsorbed water and volatile surface species, whereas the major mass loss at higher temperature corresponds to carbon and Ti_3C_2 oxidation.*

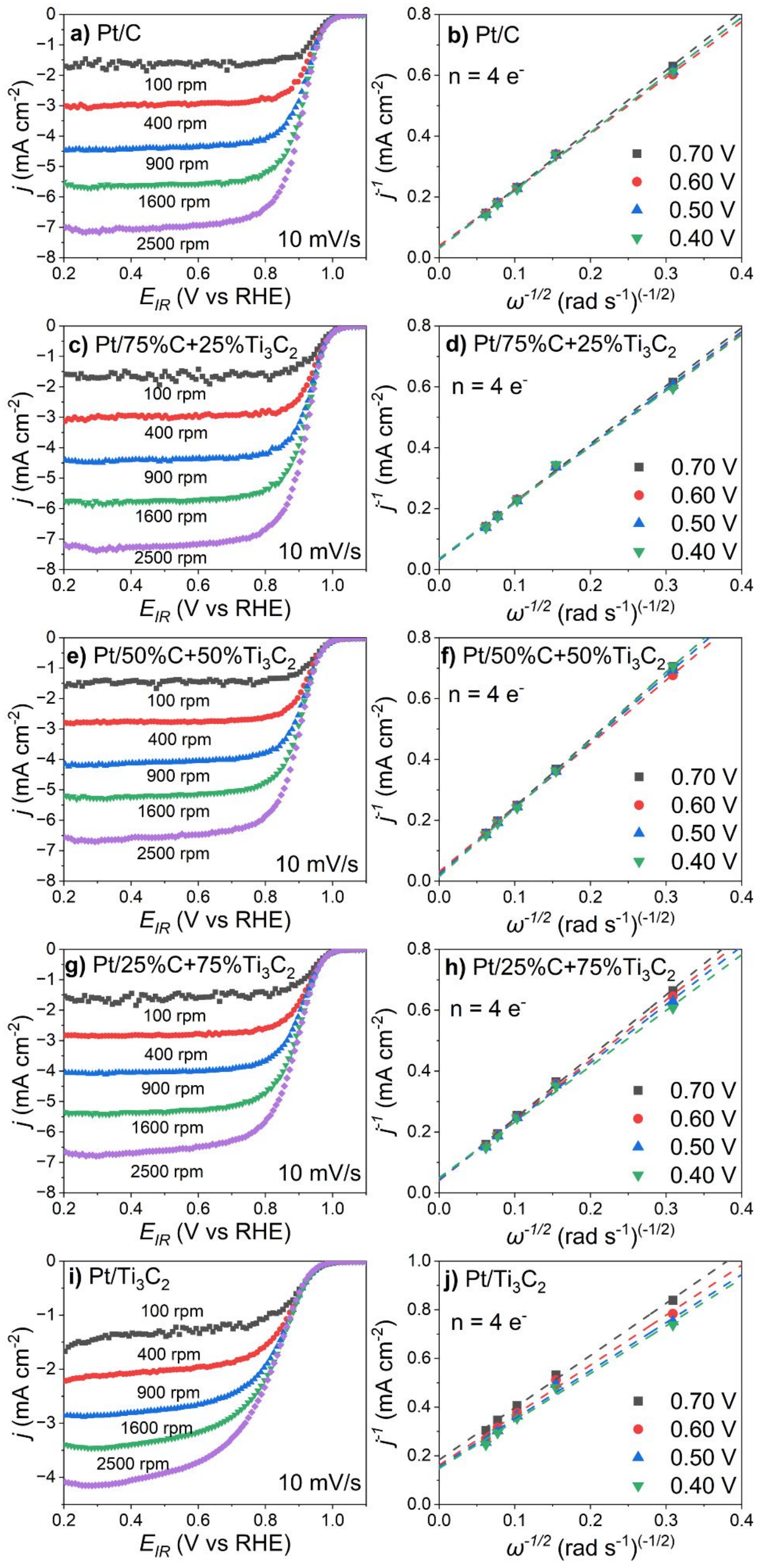

Figure SI 10: *LSV plots of the samples with changing rotation speed and their corresponding Koutecký - Levich plots.*

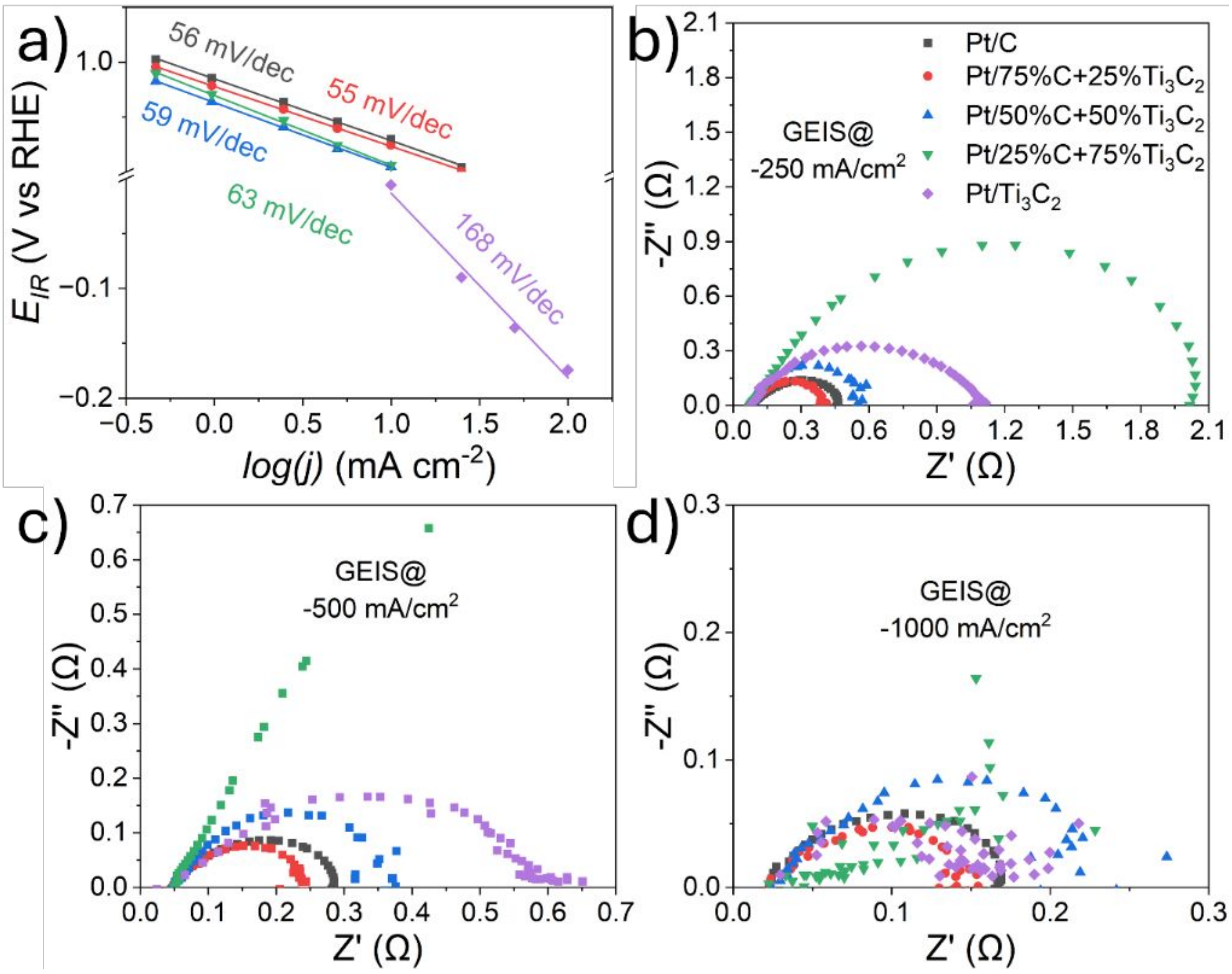

Figure SI 11: *Gas-diffusion half-cell ORR in N_2-saturated 1.0 M $HClO_4$ at room temperature. a) Tafel slopes from kinetic currents. b-c-d) GEIS Nyquist at -250 mA/cm², -500 mA/cm², and -1000 mA/cm², respectively.*

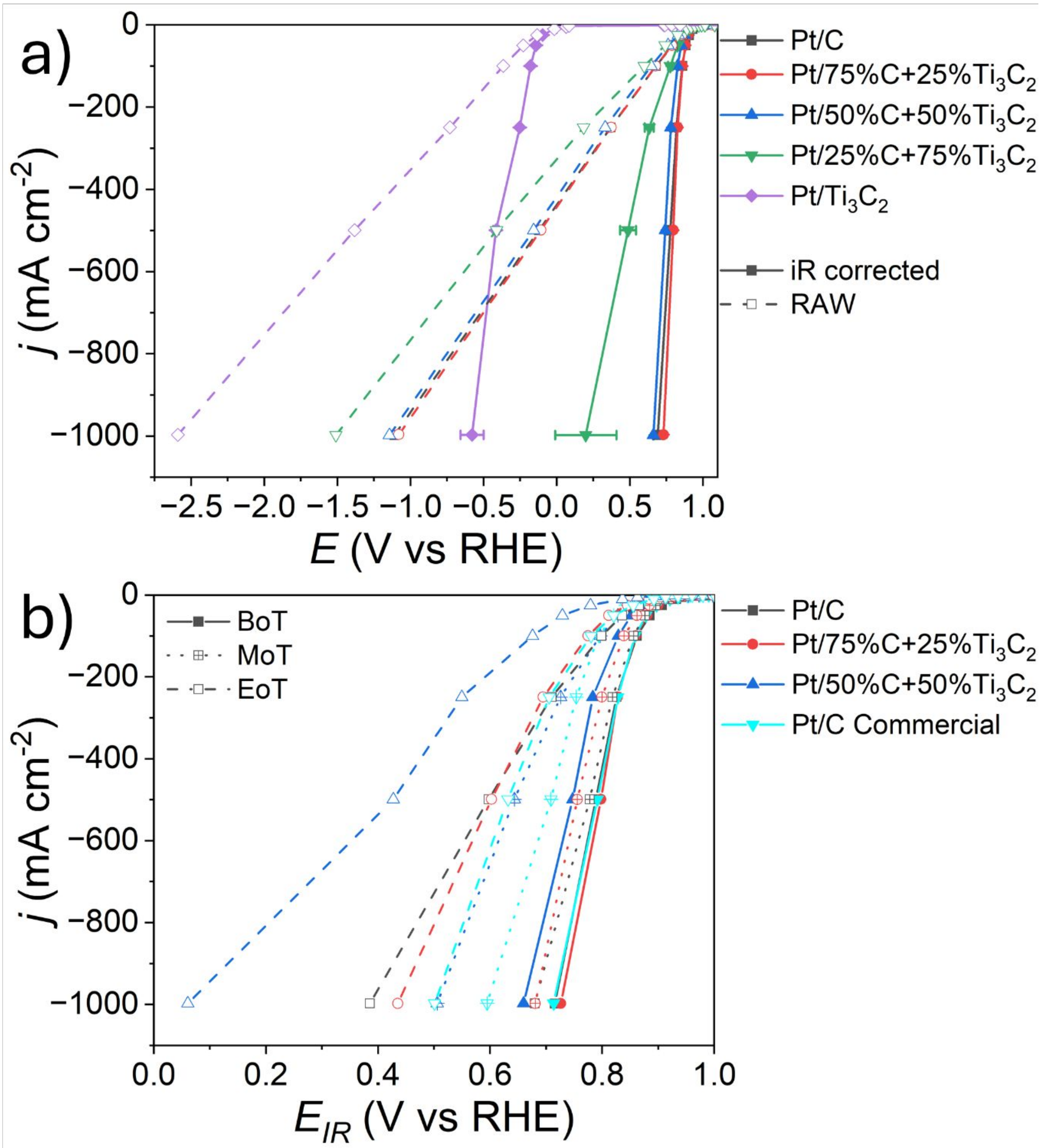

Figure SI 12: *a) iR corrected and RAW polarization curves of the samples. b) iR corrected polarization curves of the samples for support stability test, including 15 000 cycle performance and commercial Pt/C.*

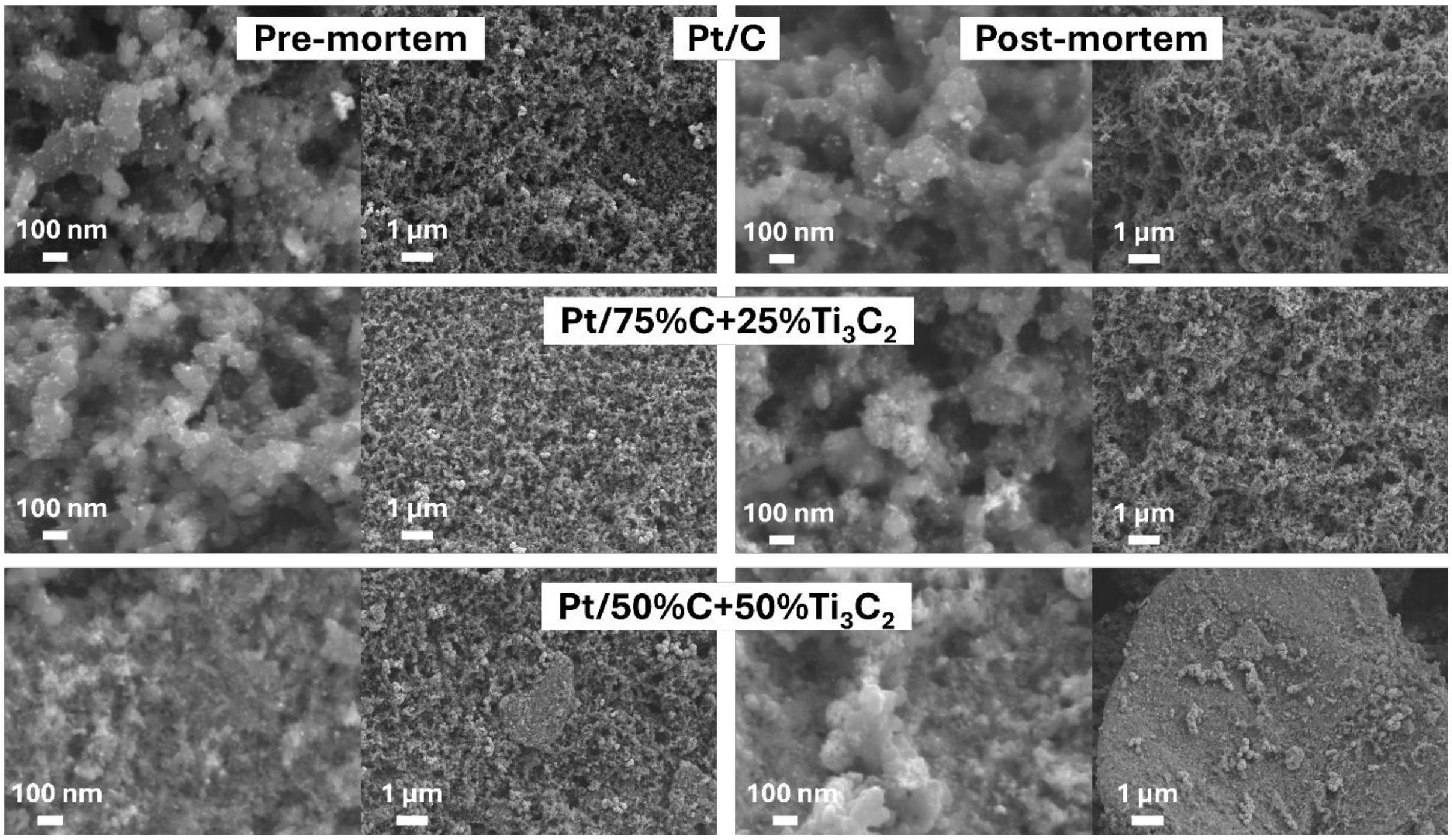

Figure SI 13: *SEM of catalyst coated carbon-cloth electrodes before and after stability testing: morphology unchanged, continuous catalyst layer, no new cracks or delamination; representative low/high magnifications shown.*

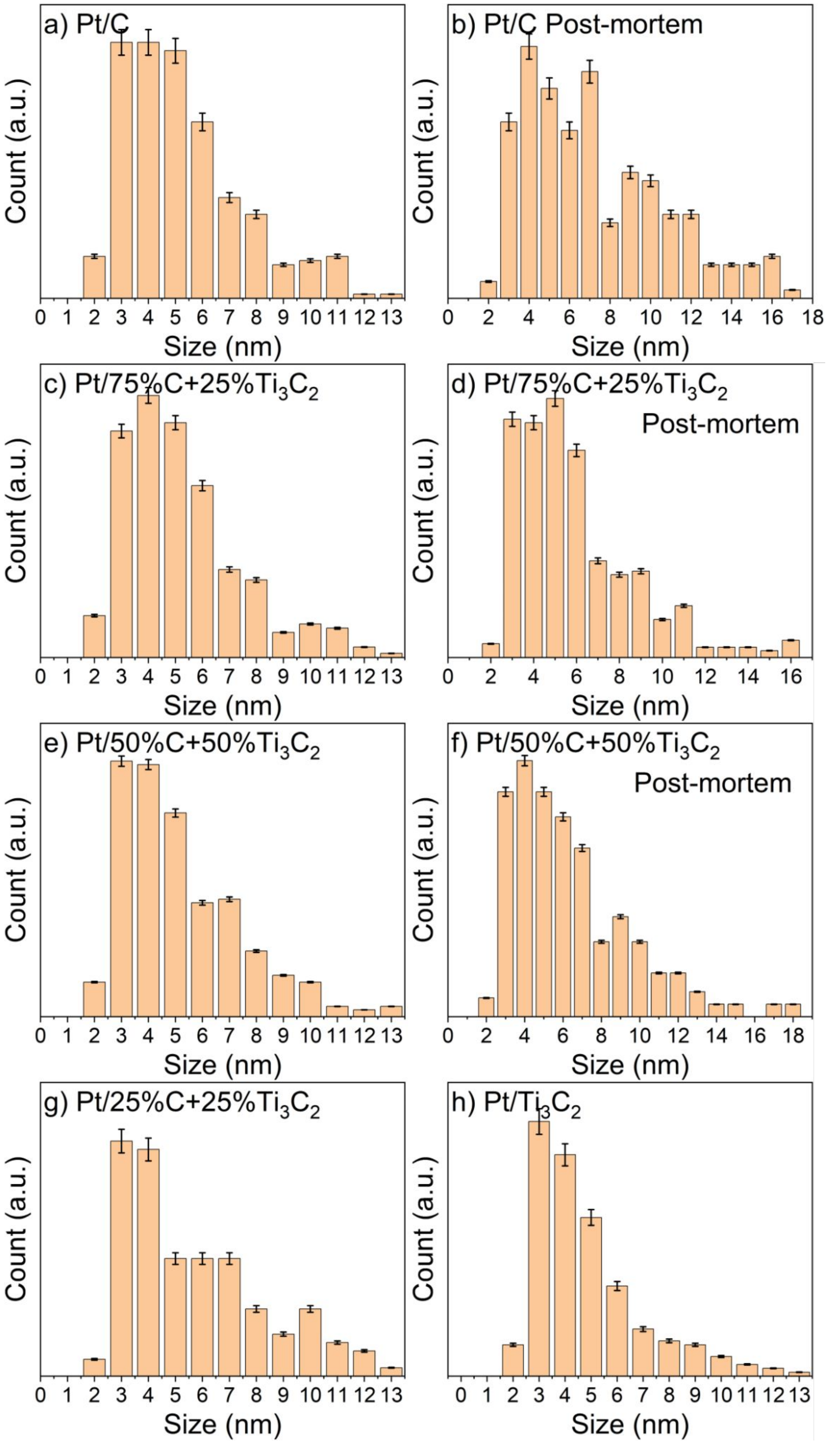

Figure SI 14: *Pt NP distribution across the samples.*

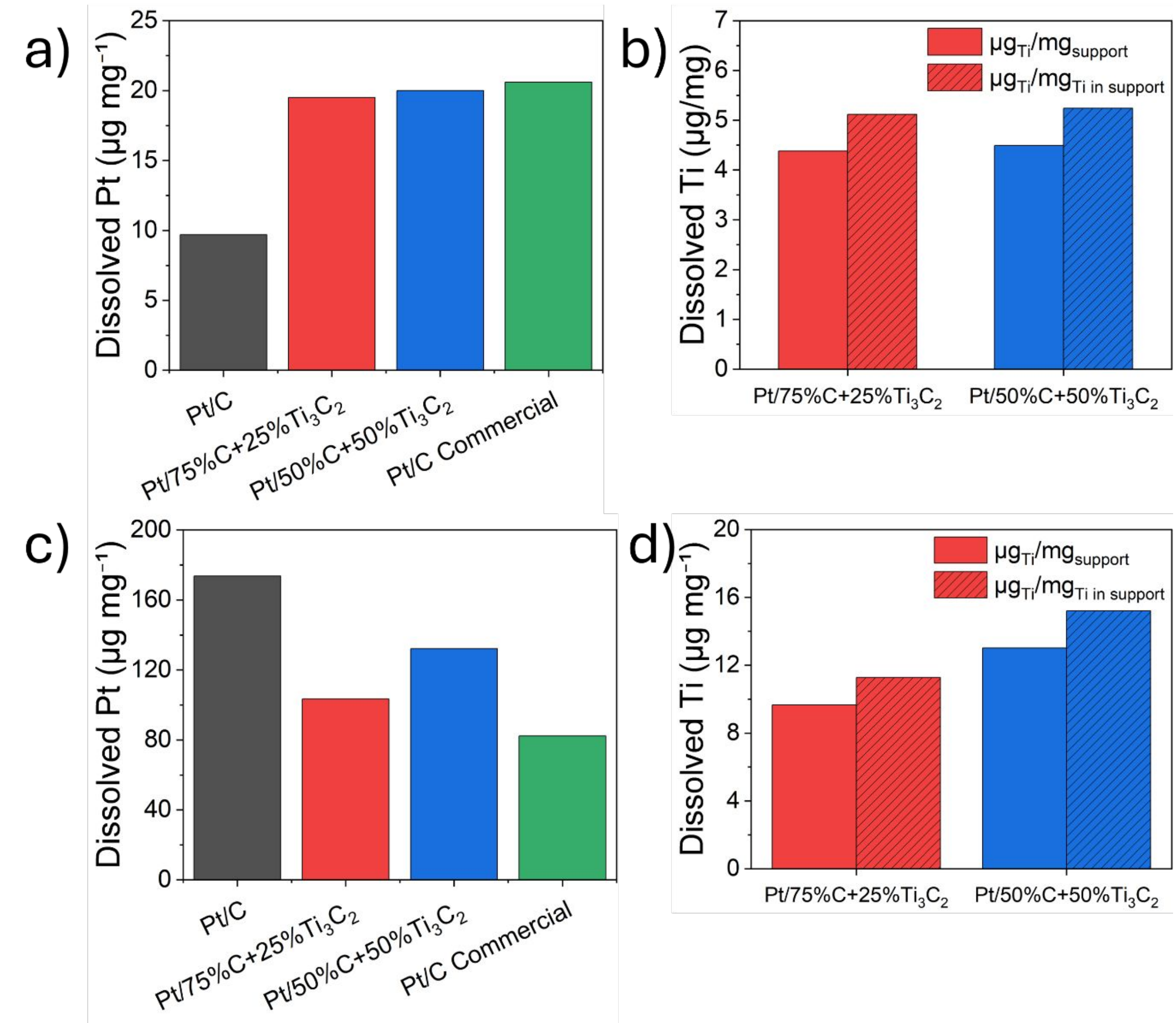

Figure SI 15: *Dissolved Pt and Ti normalized to initial Pt and support contents after the accelerated stress tests. a) Dissolved Pt after Pt AST. b) Dissolved Ti after Pt AST. c) Dissolved Pt after support AST. d) Dissolved Ti after support AST.*

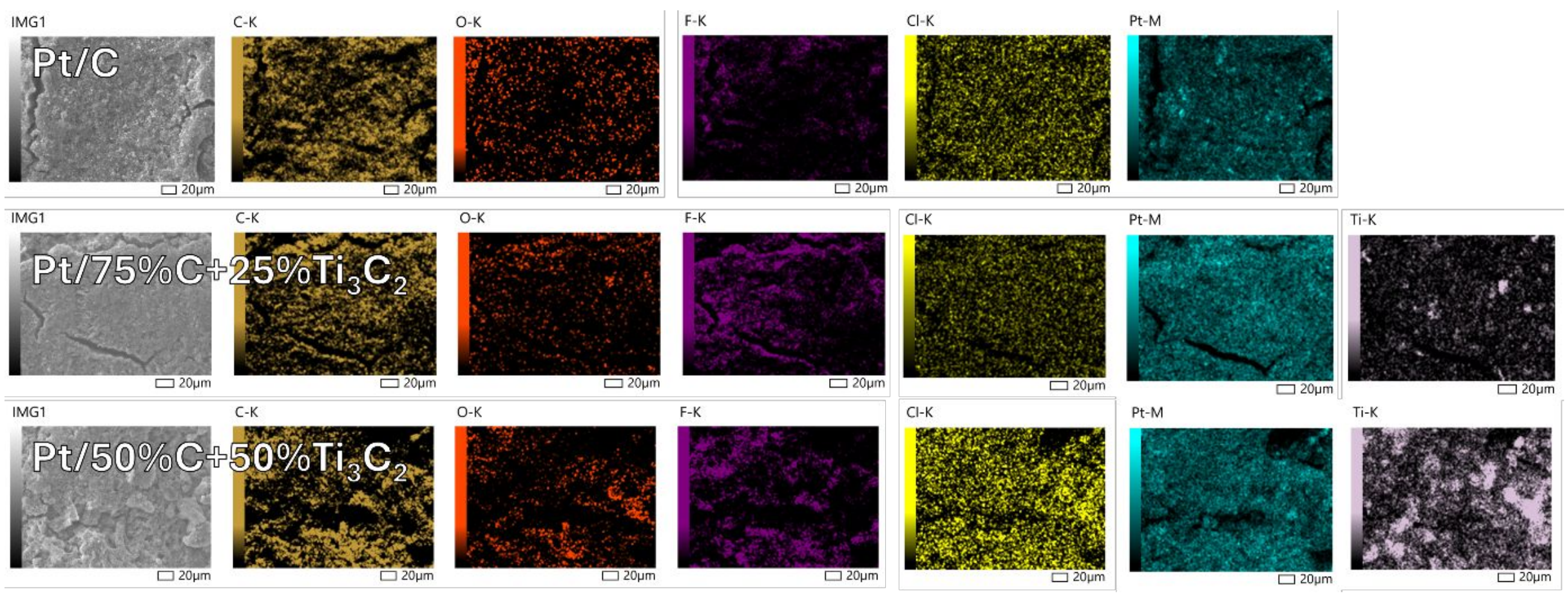

Figure SI 16: *EDX mapping of the post-mortem electrodes after support AST.*

Table SI 3: *Literature comparison of the similar Pt/Ti_3C_2 or Pt/TiO_X studies for acidic ORR. MA and SA values in the table are chosen from their best samples.*

Study	Catalyst	Test Method	MA (A/mg)	SA (mA/cm2)	Pt-AST Method	Support-AST Method
This Work	Pt/75%C+25%Ti_3C_2	• RDE<br>• GDE Half-cell	0.143 (0.9 V, RDE)<br>0.133 (0.85 V, GDE)	0.370 mA/cm2<br>(0.9 V, RDE)	**GDE Half-cell**, 0.6-0.95 V, 30k square wave cycles, 3 s each, 4% loss at 1 A/cm2, Pt 5.2->5.9 nm (TEM), Pt loss 19.5 µg/mg	**GDE Half-cell**, 1.0-1.5 V, 30k square wave cycles, 3 s each, 291 mV loss at -1 A/cm^2, Pt loss 10.3%, Ti loss 1.13%
Chen et al.[10]	Pt/TiO_X-Vo	• RDE	-	-	**RDE**, 0.6-1.15 V, 10k CV cycles, 50 mV/s, ECSA/MA loss 39.7% vs Pt/C 44.8%/46.9%; Pt leach ~4 wt%	-
Xu et al.[11]	Pt/1CNT:1$Ti_3C_2T_X$	• RDE<br>• Single-cell MEA<br>• 22-cell stack	0.163 (0.9 V, RDE)	0.147 (0.9 V, RDE)	**RDE**, 1.25-0 V, 2k CV cycles, 10 mV/s, ECSA loss 6% vs 27% for Pt/C	-
Zhang et al.[12]	Pt/2C:1Ti_3C_2	• RDE	0.110 (0.9 V, RDE)	0.377 (0.9 V, RDE)	**RDE**, 0.6-1.0 V, 30k CV cycles, 100 mV/s, 1.73% MA loss	**RDE**, 1.0-1.5 V, 30k CV cycles, 100 mV/s
Liu et al.[13]	Pt/TiO_2/C	• RDE<br>• Single-cell MEA	-	-	**RDE**, -0.25-1.0 V, 1k CV cycles, 50 mV/s, ECSA retained 75.6% vs 42.6% Pt/C	**MEA**, 0.6-1.2 V, 3k CV cycles, 50 mV/s, 18 mV at -1 A/cm^2
Zhang et al.[14]	Pt/$Ti_3C_2T_X$	• RDE	-	-	**RDE**, 0-1.2 V, 1.5k CV cycles, 200 mV/s, current retention 89.9-93.6% vs 70.6-85.6% Pt/C	-

References

[1] M. Lim, M. Khalid, S. Shahgaldi, and X. Li, “Development of Non-Spherical Platinum Nanoparticles on Carbon Supports for Oxygen Reduction Reaction,” *Catalysts*, vol. 13, no. 10, 2023, doi: 10.3390/catal13101322.

[2] M. P. Seah and W. A. Dench, “Quantitative electron spectroscopy of surfaces: A standard data base for electron inelastic mean free paths in solids,” *Surf. Interface Anal.*, vol. 1, no. 1, pp. 2–11, 1979, doi: 10.1002/sia.740010103.

[3] J. J. Yeh and I. Lindau, “Atomic subshell photoionization cross sections and asymmetry parameters: $1 \le Z \le 103$,” *At. Data Nucl. Data Tables*, vol. 32, no. 1, pp. 1–155, 1985, doi: 10.1016/0092-640X(85)90016-6.

[4] F. Amargianou *et al.*, “Nanoscale Surface and Bulk Electronic Properties of Ti3C2Tx MXene Unraveled by Multimodal X-Ray Spectromicroscopy,” *Small Methods*, vol. 8, no. 12, pp. 1–9, 2024, doi: 10.1002/smtd.202400190.

[5] N. Sharma *et al.*, “Nanoscale chemical imaging of pseudocapacitive charge storage in MXenes,” *Energy Environ. Sci.*, 2025, doi: 10.1039/d5ee05809k.

[6] K. Ehelebe *et al.*, “Benchmarking Fuel Cell Electrocatalysts Using Gas Diffusion Electrodes: Inter-lab Comparison and Best Practices,” *ACS Energy Lett.*, vol. 7, no. 2, pp. 816–826, 2022, doi: 10.1021/acsenergylett.1c02659.

[7] L. F. Pearson, “Fuel Cell Technologies Office,” *Organ. Energy Ind.*, pp. 23–54, 2017.

[8] S. Polani *et al.*, “Oxygen Reduction Reaction Activity and Stability of Shaped Metal-Doped PtNi Electrocatalysts Evaluated in Gas Diffusion Electrode Half-Cells,” *ACS Appl. Mater. Interfaces*, vol. 16, no. 39, pp. 52406–52413, 2024, doi: 10.1021/acsami.4c11068.

[9] D. A. Cullen *et al.*, “New roads and challenges for fuel cells in heavy-duty transportation,” *Nat. Energy*, vol. 6, no. 5, pp. 462–474, 2021, doi: 10.1038/s41560-021-00775-z.

[10] Y. Chen, J. Chen, J. Zhang, Y. Xue, G. Wang, and R. Wang, “Anchoring Highly Dispersed Pt Electrocatalysts on TiOx with Strong Metal-Support Interactions via an Oxygen

Vacancy-Assisted Strategy as Durable Catalysts for the Oxygen Reduction Reaction," *Inorg. Chem.*, vol. 61, no. 12, pp. 5148–5156, 2022, doi: 10.1021/acs.inorgchem.2c00329.

[11] C. Xu *et al.*, "MXene (Ti3C2Tx) and Carbon Nanotube Hybrid-Supported Platinum Catalysts for the High-Performance Oxygen Reduction Reaction in PEMFC," *ACS Appl. Mater. Interfaces*, vol. 12, no. 17, pp. 19539–19546, 2020, doi: 10.1021/acsami.0c02446.

[12] Z. Zhang *et al.*, "Sandwich-Structured MXene/Carbon Hybrid Support Decorated with Pt Nanoparticles for Oxygen Reduction Reaction," *ACS Appl. Energy Mater.*, vol. 5, no. 12, pp. 14957–14965, 2022, doi: 10.1021/acsaem.2c02479.

[13] X. Liu, J. Chen, G. Liu, L. Zhang, H. Zhang, and B. Yi, "Enhanced long-term durability of proton exchange membrane fuel cell cathode by employing Pt/TiO2/C catalysts," *J. Power Sources*, vol. 195, no. 13, pp. 4098–4103, 2010, doi: 10.1016/j.jpowsour.2010.01.077.

[14] C. Zhang, B. Ma, Y. Zhou, and C. Wang, "Highly active and durable Pt/MXene nanocatalysts for ORR in both alkaline and acidic conditions," *J. Electroanal. Chem.*, vol. 865, 2020, doi: 10.1016/j.jelechem.2020.114142.
